# Iridium trihydride and tetrahydride complexes and their role in catalytic polarisation transfer from *para*hydrogen to pyruvate†

**DOI:** 10.1039/d4sc06138a

**Published:** 2024-12-12

**Authors:** Ben. J. Tickner, Simon B. Duckett

**Affiliations:** a Centre for Hyperpolarisation in Magnetic Resonance, University of York Heslington YO10 5NY UK; b Department of Chemistry, University of York Heslington YO10 5DD UK simon.duckett@york.ac.uk

## Abstract

This work details how the unusual iridium tetrahydride [Ir(H)_4_(IMes)(sulfoxide)]Na and trihydride [Ir(H)_3_(IMes)(sulfoxide)_2_] can be formed in a solvent dependent reaction of [IrCl(COD)(IMes)] with sulfoxide (dimethyl or methylphenyl), base, and H_2_. In the case of dimethyl sulfoxide, the four hydride ligands of the tetrahydride are equivalent, and the IMes and sulfoxide ligands mutually *trans*. However, for phenyl methyl sulfoxide, this isomer of the tetrahydride forms alongside its *cis* counterpart where the remarkable symmetry breaking effect of the sulfoxide leads to it presenting four chemically distinct hydride ligands. These products and their ligand arrangements are characterised and the reaction pathways leading to their formation probed using NMR spectroscopy and *para*hydrogen-hyperpolarised methods. Subsequently, they form as previously unidentified low concentration by-products in the important SABRE-catalysed hyperpolarisation of pyruvate, and their concentration should be minimised for efficient polarisation transfer.

## Abstract 2

This work details formation of unusual [Ir(H)_4_(IMes)(sulfoxide)]Na and [Ir(H)_3_(IMes)(sulfoxide)_2_] from Ir(i) precursors, alongside binuclear products. Their role in the catalytic transfer of magnetisation from *para*hydrogen is investigated.

## Graphical abstract

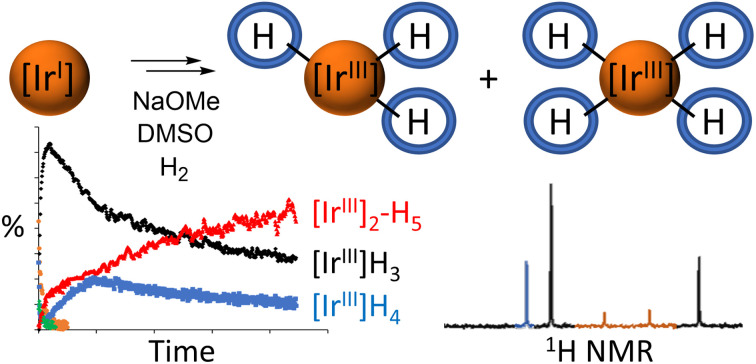

## Introduction

Metal hydride complexes play a key role in industrial catalysis, fine-chemical manufacture, and materials chemistry.^1^ Some of the more unusual examples of metal hydrides are reflected in {RuH_4_} species that commonly exist in dihydride–dihydrogen arrangements,^2^ and react to form dimers in the absence of coordinating ligands.^3,4^ These species are readily formed by the reaction of a chloride precursor with H_2_ and MOH [M = K or Na]. Interestingly, their deprotonation affords anionic trihydride complexes like [RuH_3_(PPh_3_)_3_]M that are useful building blocks for complex structures,^5^ with 18-crown-6-ether enabling structural studies that confirm their facial hydride ligand arrangement.^6^ In contrast related [OsH_4_(PPh_3_)_3_] is a classical Os(iv) tetrahydride,^7^ and while *fac*-[OsH_3_(PPh_3_)_3_]^−^ is itself highly reactive,^8^ it can be stabilised by ion pairing effects.^9^ For rhodium, while many trihydride complexes are known, they are normally highly reactive and their {RhH_4_} analogues are again cationic dihydride–dihydrogen species.^10,11^

Surprisingly, a wider range of iridium hydride species are known, and these exhibit both classical and non-classical interactions. Notably, pincer ligands have been used to generate neutral Ir dihydrides that now exist in equilibrium with their tetrahydride counterpart.^12,13^ Now, accessible pentahydride complexes like [Ir(H)_5_(P^*i*^Pr_3_)_2_], act as weak acids and form anionic tetrahydride Ir(iii) species in the presence of KH, with *cis* and *trans* isomers forming according to the counterion environment.^14,15^ More recently, related [IrH_4_(dppe)]K has been obtained from [IrCl(COD)(dppe)] and KO^*t*^Bu in isopropanol.^16^

The bonding and ligand mobility shown by such metal hydride complexes can be readily investigated using nuclear magnetic resonance (NMR) spectroscopy through hydride ligand chemical shifts, H–H and related coupling constants, and relaxation data.^17^ Furthermore, as hydride ligands are often associated with the activation of molecular H_2_ metal–dihydrogen complexes that retain a strong H–H bonding interaction have added significantly to our understanding of bond activation processes more generally.^17,18^

One example of catalysis that relies on these effects is highly topical reversible magnetisation transfer from a *para*hydrogen (*p*H_2_) feedstock. This is typically mediated by a metal complex that activates *p*H_2_ to unlock its latent spin order.^19–22^ Magnetisation can then spread either spontaneously,^23,24^ or *via* radiofrequency excitation,^25,26^ to other sites within the metal complex from the *p*H_2_ derived hydride ligands. Ligand dissociation then allows the catalytic build-up of a finite concentration of hyperpolarised ligand, free in solution, with the complex playing a key role in the efficiency of this catalytic process as ligand exchange rates, *J*-coupling, relaxation, and solubility all affect polarisation transfer.^27,28^

Typically, such spin polarisation transfer catalysts are iridium-derived dihydride complexes of the form [Ir(H)_2_(NHC)(substrate)_3_]Cl or [IrCl(H)_2_(NHC)(substrate)_2_], where NHC is an *N*-heterocyclic carbene.^29,30^ This process has been termed signal amplification by reversible exchange (SABRE). Significantly, experimentally invisible but theoretically predicted, {Ir(H)_2_(η^2^-H_2_)}-type reaction intermediates are thought to play a crucial role in refreshing the *p*H_2_-derived singlet order within the active catalyst that is needed for SABRE,^29,31^ and hydride complexes derived from this family play a key role in other metal-catalysed pathways such as hydrogenation, hydroformylation, and deuterium isotope exchange.^32,33^

**Fig. 1:**
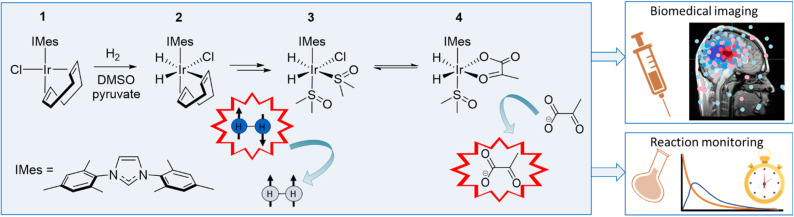
Mechanism leading to the successful hyperpolarisation of pyruvate by SABRE. The reaction of 1 with H_2_, DMSO and sodium pyruvate in methanol-d_4_ forms an equilibrium mixture of 3 and 4*via*2. Reversible exchange of *p*H_2_ and pyruvate then dramatically enhance the ^13^C NMR signals of free pyruvate. Such enhanced signals have been used in applications that include biomedical imaging and reaction monitoring as eluded to in the cartoons shown.

In this work, we use NMR to rationalise the detection of a series of iridium *tri* and *tetra* hydride species that form when the common SABRE catalyst precursor [IrCl(COD)(IMes)] (COD = *cis–cis*-1,5-cyclooctadiene and IMes = 1,3-bis(2,4,6-trimethyl-phenyl)imidazole-2-ylidene) reacts with sulfoxide, base, and H_2_. These unstable species are reflective of [Ir(H)_3_(IMes)(sulfoxide)_2_] and [Ir(H)_4_(IMes)(sulfoxide)]Na, and characterised using a combination of detailed NMR studies and *para*hydrogen-hyperpolarised NMR. We highlight routes to their formation and comment on their reactivity, which leads to the eventual detection of a number of binuclear reaction products. We also rationalise the role these complexes play in the SABRE hyperpolarisation of pyruvate as first reported in 2019 (Fig. 1).^34^ Given the proven utility of pyruvate as a viable molecular imaging probe of cancer, such studies are highly warranted. Furthermore, since this initial report utilising SABRE, pyruvate polarisation levels have been optimised^35–37^ to the point *in vivo* biomedical imaging is possible.^38^

As shown in Fig. 1, the addition of H_2_ (3 bar) to methanol-d_4_ solutions containing [IrCl(COD)(IMes)] (1) (5 mM), DMSO (25 mM) and sodium pyruvate (30 mM) is complex and proceeds by the initial formation of [IrCl(H)_2_(COD)(IMes)] (2), before further reaction leads to an equilibrium mixture of [IrCl(H)_2_(IMes)(DMSO)_2_] (3) and [Ir(H)_2_(κ^2^-pyruvate)(IMes)(DMSO)] (4).^34,39^4 exists in two dominant isomeric forms, according to the orientation of the pyruvate and hydride ligands, with optimal SABRE hyperpolarisation of pyruvate stemming from the isomer where these ligands lie in the same plane. Highly reactive [IrCl(H)_2_(IMes)(DMSO)_2_], 3, is therefore present in these reaction mixtures, and it forms when the reaction is performed without sodium pyruvate.^34,39^3 has been suggested to play a key role in *p*H_2_ incorporation during such studies.^39^

These early reports addressed the identities of the major products, 3 and 4, detected during SABRE.^35,37,38,40,41^ Other minor hydride-containing species prove visible to NMR during these studies, with X-ray crystal structures resulting for several sulfur-bridged iridium dimers that form at long reaction times. These have been linked to a drop in catalyst activity over time.^42,43^ However, the identity and the role of the minor reaction products has not yet been resolved due to the fact their NMR signals appear only transiently. This stems from the use of methanol-d_4_ as the solvent, which leads to the rapid deuteration of any hydride ligands, and this precludes their spectral mapping. Accordingly, we begin this work by studying the reactivity of [IrCl(COD)(IMes)] 1 with sulfoxide, and H_2_ in methanol-d_3_ and detail some surprising discoveries.

## Results and discussion

### Formation of [Ir_2_(Cl)(μ_2_-Cl)(H)_3_(μ_2_-H)(IMes)_2_(DMSO)_2_] (5 and 5′)

When solutions containing 1 and DMSO are reacted with H_2_ in methanol-d_3_ they lead to the rapid formation of 3, as expected. 3 yields two very broad hydride ligand NMR signals due to rapid sulfoxide and H_2_ exchange at 298 K. Its detection when such studies are completed in methanol-d_4_ at 298 K by NMR is made even more challenging by the fact its hydride ligand sites become rapidly deuterated as CD_3_OH forms.

**Scheme 1:**
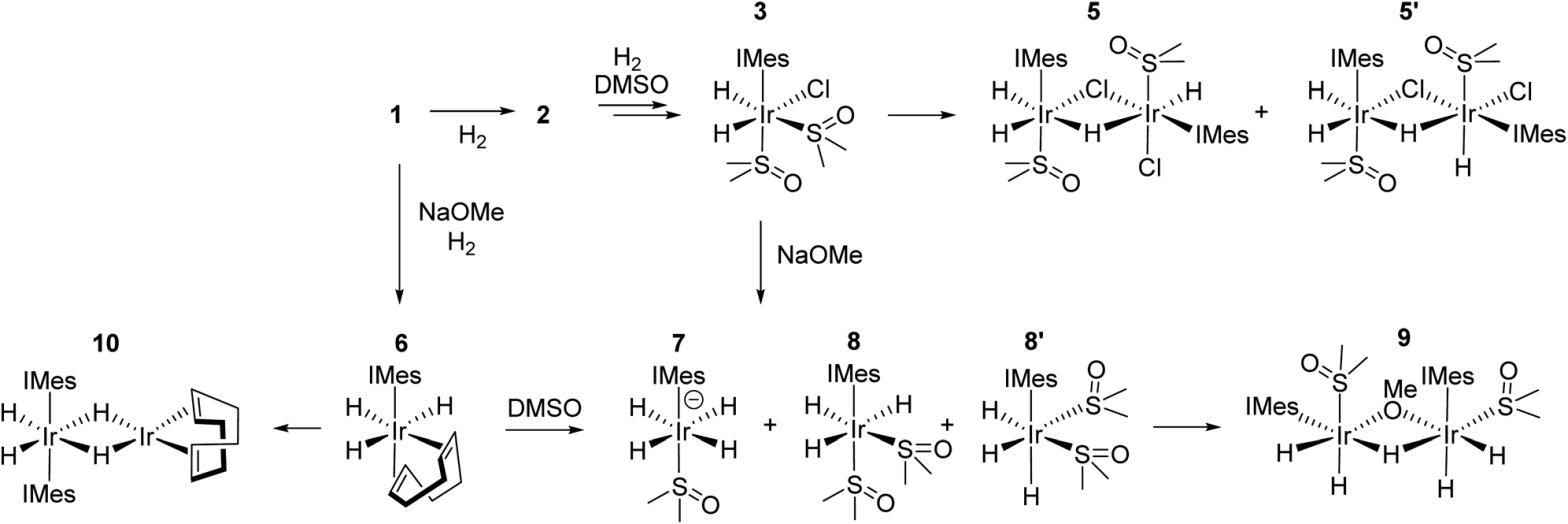
Reaction pathways revealed through this work build from known 1–3, 6 and 10, with some of these species reported to play a role in iridium-mediated polarisation transfer from *p*H_2_ to pyruvate. These complexes were detected here, alongside previously unreported 5, 5′, 7, 8, 8′, and 9. The formation of 6–9 are facilitated by NaOMe, added specifically or formed indirectly when sodium pyruvate is utilised in methanol.

However, in the methanol-d_3_ solution the ready detection of a range of other hydride ligand signals besides those of 3 proved possible. The most notable of these signals appear at *δ* = −15.79, −16.06, −24.80, and −30.66, and possess equivalent signal intensities. They were assigned to the dimer [Ir_2_(Cl)(μ_2_-Cl)(H)_3_(μ_2_-H)(IMes)_2_(DMSO)_2_], 5 of Scheme 1 by a series of complex NMR procedures (see ESI, Section S1†). A second set of much weaker signals appear at *δ* = −16.90, −18.82, −20.55, and −22.11 were assigned to a minor isomer (5′) of 5 that exhibits a different ligand arrangement (see ESI, Section S1†). 5 and 5′ likely arise from the dimerization of 3, and differ from the literature reported sulfur-bridged dimers that have been reported to form at very long reaction times.^42,43^

### Formation of the tetrahydride [Ir(H)_4_(DMSO)(IMes)] (7) and trihydride [Ir(H)_3_(DMSO)_2_(IMes)] (8 and 8′)

Recently, the unstable trihydride complex [Ir(H)_3_(COD)(IMes)] (6) was characterised as a minor reaction product alongside dominant [Ir(H)_2_(IMes)(pyridine)_3_]Cl when the weak base pyridine and H_2_ react with 1 in methanol. The proportion of 6 was found to increase when NaOMe was added to methanol in agreement with its formation *via* deprotonation of the unseen intermediate [Ir(H)_2_(η^2^-H_2_)(COD)(IMes)].^44^ Consequently, methanol-d_3_ solutions containing DMSO and a 10-fold excess of NaOMe relative to 1 (without pyruvate) were examined here. Halide substitution was observed to create an equilibrium mixture of 1, [Ir(OCH_3_)(COD)(IMes)] and [Ir(OH)(COD)(IMes)] (from adventitious water). The displacement of chloride being incomplete, with these complexes existing in a ratio of 2.6 : 2.7 : 1 respectively at 298 K; notably no signal for [Ir(DMSO)(COD)(IMes)]Cl was observed in accordance with the weak nature of the iridium sulfoxide interaction.

**Fig. 2:**
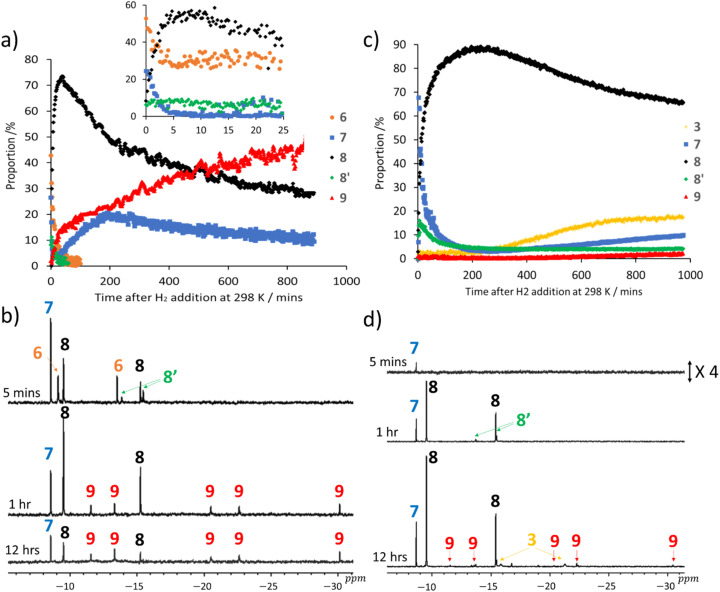
(a) Reaction time course chemical speciation plot for the changes that take place after H_2_ (3 bar) is added to 1 (5 mM), DMSO (25 mM) and NaOMe (50 mM) in methanol-d_3_ at 298 K. Note, the signals for complexes 8′ and 6 disappear after 60 and 100 minutes respectively. The inset chart shows an expansion of the first 25 minutes of this profile for a sample that was prepared in a dry ice/acetone bath prior to placing it into the NMR spectrometer at 298 K where data acquisition was started. This process allows the conversion to 6–9 to be more precisely monitored. (b) Typical hydride spectral regions extracted from the ^1^H NMR spectra used to create chart (a) at the indicated times after H_2_ addition. (c) Analogous reaction time course data collected after H_2_ (3 bar) is added to 1 (5 mM), DMSO (25 mM) and NaOMe (50 mM) in DCM-d_2_ at 298 K. (d) Typical hydride regions of the corresponding ^1^H NMR spectra used to assemble chart (c), at the indicated times after H_2_ addition.

Surprisingly, the addition of H_2_ to this solution at 298 K led to the rapid formation of three hydride-containing species. These species yielded paired signals at *δ* = −9.10 and −13.49, a unique signal at *δ* = −8.58, and paired signals at *δ* = −9.49 and −15.24 in the associated ^1^H NMR spectra. The three species initially appeared to exist in a ratio of 2.7 : 1.7 : 1 respectively, although they change with reaction time as detailed in Fig. 2a.

Initially, those for unstable [Ir(H)_3_(COD)(IMes)] (6) are dominant (Fig. 2a) with the unique hydride ligand signal, at *δ* = −8.58, being attributed to [Ir(H)_4_(DMSO)(IMes)]Na, 7 on the basis of its NMR data. This included peak integration and NOE analysis (see ESI, Section S2† for characterisation details). The evolution of the intensity of its hydride signal as a function of reaction time proved complex in this data recorded at 298 K, with its initial high intensity falling, before increasing again and then falling (Fig. 2a). This suggests there are two routes to its formation.

The NMR characterisation of 8 was secured at 243 K as the trihydride [Ir(H)_3_(DMSO)_2_(IMes)] (8) of Scheme 1. Its hydride ligand signals appear in a 2 : 1 ratio, with NOE studies confirming their meridional arrangement, alongside interactions to bound IMes and two inequivalent DMSO ligands (see ESI, Section S2† for characterisation details). Initially, 8 coexists with isomeric 8′, whose three hydrides are arranged in a facial arrangement. Its signals are observed in a 1 : 2 ratio at *δ* = −13.85 and −15.45 (see ESI, Section S2† for characterisation details), but their intensity is always low and are no longer evident after 45 minutes of reaction at 298 K. Consequently, it must form separately from 8 and the two species are not in equilibrium. After a few hours at room temperature, the ratio of 7 and 8 remains similar, suggesting that while these two species initially form in different pathways, they equilibrate slowly.

At long reaction times weak signals for 5 and 5′ appear that account for just a few % of the reaction products. However, signals for 9 at *δ* = −11.56, −13.30, −20.48, −22.61, and −30.11 also appear and these are much more substantial. They are assigned to the pentahydride dimer [Ir_2_(H)_4_(μ_2_-H)(μ_2_-OCD_3_)(DMSO)_2_(IMes)_2_] (see ESI, Section S3†). This product again likely arises from a reaction of 3, but this time with 8 or 8′ and methoxide rather than with a second molecule of 3. It is worth noting that after 15 hours of reaction the total hydride ligand signal intensity accounts for just 75% of the original iridium concentration, a situation which is not surprising give the long term formation of sulfide derived dimers.^44^

When the analogous reaction was monitored in DCM-d_2_ at 298 K (Fig. 2c) the formation of 7, 8, 8′ and 9 was again revealed. 8 now clearly dominates (Fig. 2d) the products formed in this solvent and the hydride ligand signals for 6 are very challenging to discern, with its proportion never exceeding a few %. In contrast, signals for 3 are clearly visible and grow in proportion at longer time points. Furthermore, 9 no longer forms in significant amounts as there is now significantly less methoxide available to create the necessary bridging ligand.

### Reaction pathways leading to [Ir(H)_4_(DMSO)(IMes)] (7) and trihydride [Ir(H)_3_(DMSO)_2_(IMes)] (8 and 8′)

In order to probe these reactions in more detail, a series of control measurements were completed. The first of these involved reacting 1 with H_2_ and DMSO in methanol-d_3_ to form a mixture of 3 and 5 as detailed earlier. This speciation changed immediately upon adding NaOMe to the solution as ^1^H NMR signals for 7 and 8 rapidly replaced those of 3 and 5 (see ESI, Section S4†). A second control measurement involved the initial conversion of 1 into 6*via* NaOMe addition to 1 in methanol-d_3_ under H_2_ at 253 K. This reaction also leads to formation of known [Ir_2_(H)_2_(μ^2^-H)_2_(η^2^–η^2^-COD)(IMes)_2_] (10) as a minor product.^44^ Once all of the 1 present was consumed, DMSO was added to the solution at 298 K and NMR monitoring commenced. Under these conditions, 6 proved to rapidly convert into both 7 and 8, with the later ultimately dominating.

Collectively, these results confirm that 3, 5 and 6 react independently to yield 7 and 8, which have already been stated to equilibrate. Furthermore, the reaction time-course data (Fig. 2a and c) confirm the existence of two pathways to 7 as its hydride ligand signal intensity, both in methanol-d_3_ and DCM-d_2_, exhibit two maxima. Literature examples of {IrH_3_} and {IrH_4_} species form by H_2_ addition to {IrH} and {IrH_2_} precursors respectively, or involve base-driven deprotonation of {IrH_4_} and {IrH_5_} species.^12–15^ Hence, routes to 7*via* deprotonation of undiscerned {Ir(H)_5_} and 8*via* H_2_ loss from {Ir(H)_5_}, or deprotonation of hydride dihydrogen intermediates formed from 3 and 6 are possible.

### Effect of deuteration on hydride ligand signals of [Ir(H)_4_(DMSO)(IMes)] (7) and [Ir(H)_3_(DMSO)_2_(IMes)] (8)

**Fig. 3:**
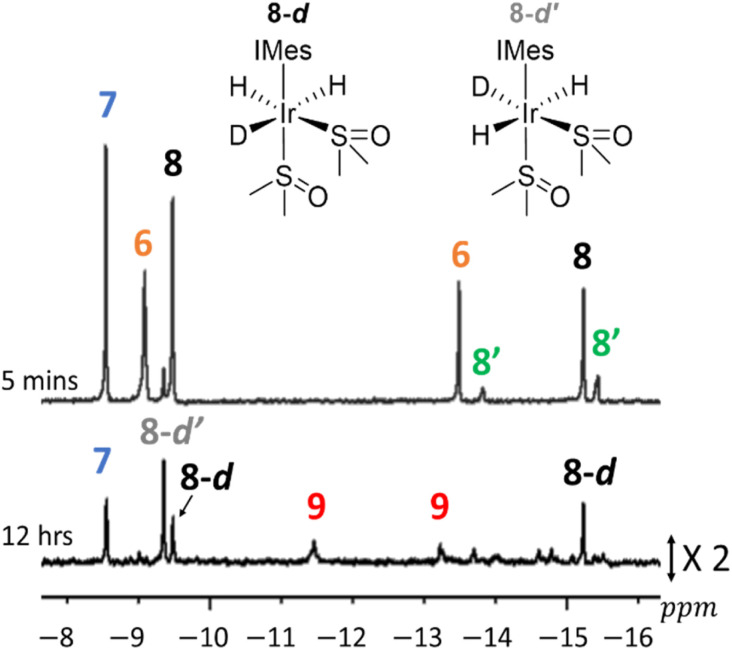
Hydride regions taken from ^1^H NMR spectra recorded at the indicated times after H_2_ (3 bar) is added to 1 (5 mM), DMSO (25 mM) and NaOMe (50 mM) in methanol-d_4_ at 298 K. Unassigned signals could result from isotopologues of 9 and other binuclear products.

As indicated earlier, when these reactions are performed in methanol-d_4_, hydride ligand exchange with the methoxyl deuterium occurs. This is readily evident as when 8 is formed initially in methanol-d_4_, the H_3_ form dominates before two regioisomers, 8-d and 8-d′, of its mono ^2^H-labelled counterpart result depending on which site contains deuterium (Fig. 3). For 8-d, the deuterium label is incorporated into the site *trans* to hydride, and hence it yields inequivalent hydride ligand signals at essentially the same chemical shifts as those of 8, but now in a 1 : 1 ratio. In contrast, its regioisomer 8-d′ contains two equivalent hydride ligands as the deuterium label lies *trans* to DMSO, and yields a single hydride signal at *δ* = −9.37 (Fig. 3a). Subsequently these signals disappear as 8 becomes fully deuterated in addition to reacting to form deuterated analogues of 9 and its binuclear counterparts.

The effect of deuterium exchange on the hydride ligand signals for 3 and 7 is, however, much less obvious, although these species are clearly deuterated; the resonances for 3 broaden, are less intense, and more challenging to discern. Those of 7 in contrast no longer integrate to four when compared to the IMes ligand signals. Furthermore, the diagnostic signals of 5, 5′, and 9, are much harder to discern due to their formation from these deuterated precursors, and/or the fact that their hydride ligands can become deuterated *via* exchange with solvent. Consequently, in methanol-d_4_ after 15 hours reaction, *ca.* 75% of the starting hydride ligand signal intensity is lost.

### Hyperpolarised measurements to rationalise formation of [Ir(H)_4_(DMSO)(IMes)] (7) and [Ir(H)_3_(DMSO)_2_(IMes)] (8)

In order to learn more about these reactions, studies using *para*hydrogen-enhanced ^1^H NMR spectroscopy were completed.^45–53^ Accordingly, a sample containing 1 (5 mM), DMSO (25 mM) and NaOMe (50 mM) in methanol-d_3_ was exposed to *p*H_2_ at 298 K, and then shaken in a magnetic field of 65 G for 10 seconds before being rapidly placed into a 9.4 T NMR spectrometer and examined using a single-scan 45° ^1^H radio frequency pulse. This process should produce enhanced ^1^H NMR signals for hydride ligands that were previously located in the *p*H_2_ feedstock if the rate of hydrogen addition is faster than the rate of nuclear spin relaxation. Under these conditions, the enhanced hydride ligand signals may display a mixture of ALTADENA and PASADENA effects if the hydrogenation step takes place at both low field and high field respectively.^21^ However, if the resulting iridium dihydride rapidly converts to a second species then its signals may too be recorded as a hyperpolarised response.^31,44^

**Fig. 4:**
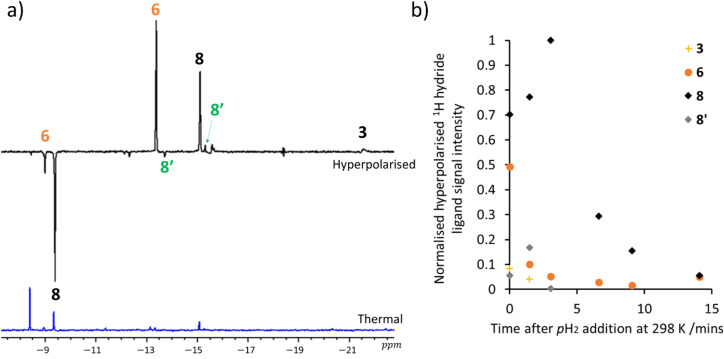
(a) Hyperpolarised single scan ^1^H NMR spectra recorded using a 45° pulse immediately after the addition of *p*H_2_ to a methanol-d_3_ solution of 1 (5 mM), DMSO (25 mM) and NaOMe (50 mM) at 298 K. Before the spectrum was recorded, the sample was shaken with *p*H_2_ for 10 seconds at 65 G to initiate PHIP. The associated time course data detailing the corresponding change in hyperpolarised signal intensity given in (b). Each data point is collected after refilling the tube with fresh *p*H_2_ and reshaking as described in (a).

Consequently, the ^1^H NMR spectrum recorded immediately after *p*H_2_ addition revealed enhanced hydride ligand signals for both 8 and 8′ with ALTADENA patterns (Fig. 4a). In contrast, those of 6 displayed strong ALTADENA behaviour, with a small PASADENA distortion. Broad enhanced hydride ligand signals for 3 with PASADENA character were also visible (Fig. 4a) that are consistent with its rapid H_2_ exchange. When further measurements are recorded after this sample is removed and shaken again with fresh *p*H_2_ for 10 seconds at 65 G the enhanced signals for 3 are no longer visible, although those of the trihydrides 6 and 8 appear, albeit with reduced intensity (Fig. 4b). Repeating this process sees the intensity of the enhanced ^1^H NMR signals decrease until they are not visible with enhancement after 1 hour of reaction. It is worth noting that other transient hydride ligand signals of similar intensity to the enhanced signals for 8′ are also discernible in the initial hyperpolarised measurement (Fig. 4a), although their identity could not be confirmed as they are only visible in hyperpolarised experiments. When the aliphatic region of these NMR spectra are examined, a PHIP enhanced signal for hydrogenated COD (cyclooctane) at *δ* = 1.5 is observed in addition to weak SABRE enhanced IMes ligand signals. These results highlight that trihydrides 6, 8, and 8′ can be created in a hyperpolarised form *via* PHIP.

Exchange spectroscopy (EXSY) studies on 8 were then conducted that revealed its H_2_ loss rate to be negligible (<4% exchange after a mixing time of 1.3 s at 298 K). Hence, it is clear that reversible H_2_ loss from 8 does not account for the PHIP response it exhibits. Furthermore, this deduction is supported by the fact that the intensity of its hyperpolarised signals decrease over the first *ca.* 15 minutes of reaction (Fig. 4b), despite the fact its concentration grows during this period (Fig. 2a). If 8 could exchange H_2_ it would be reflected in a growth of hyperpolarised signal intensity as its concentration increases. As this is not the case, its polarised response must result from the fact that it forms from a hyperpolarised precursor (*e.g.*3 or 6) with a faster formation rate than the rate of relaxation. This is consistent with the fact that 3 has already been reported to undergo rapid hydrogen exchange,^39^ while 6 has been reported to form in a hyperpolarised state, *via* deprotonation of [Ir(H)_2_(η^2^-H_2_)(COD)(IMes)] which forms after *p*H_2_ addition to 1 to form hyperpolarised 2.^44^ This hypothesis is supported by the fact that signals for hyperpolarised 3 (in the early stages of reaction) and 6 are observed throughout the time course of the hyperpolarisation experiment (Fig. 4) and the hyperpolarisation of 8 is no longer observed once 3 and 6 have been consumed. These data in turn support the previous conclusion that that 8 forms from 3 and/or 6.

Tetrahydride 7 does not display any obvious hyperpolarisation, although as its hydrides are magnetically equivalent, any hyperpolarisation is likely to be locked in a singlet state which is invisible to these NMR experiments. EXSY measurements also revealed that DMSO exchange in 7 and 8 does not occur on the NMR timescale, and that 3, 6–9 do not interconvert on this timescale.

When these hyperpolarisation experiments are repeated in DCM-d_2_, PHIP enhanced 2 and 3 are observed in the first measurement (see ESI, Section S5†). Weakly enhanced signals for 8 can then be detected a few minutes later. Collectively, these experiments confirm that the speed of reaction in DCM-d_2_ is too slow to produce strongly enhanced signals for 8.

### Breaking the symmetry of trihydrides and tetrahydrides using chiral sulfoxides

**Fig. 5:**
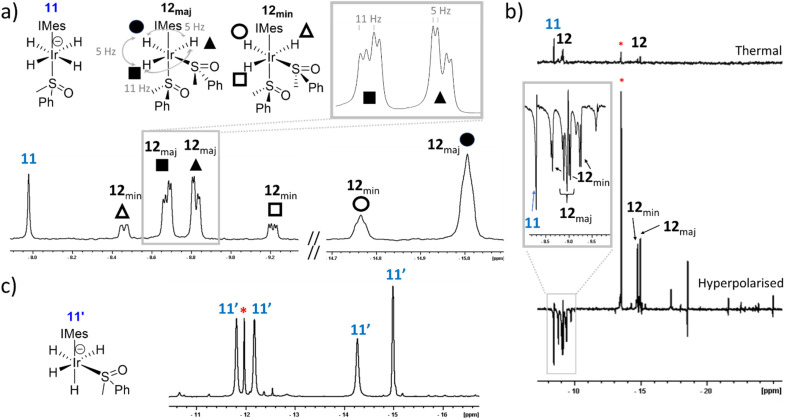
(a) Hydride regions taken from ^1^H NMR spectra recorded a few minutes after H_2_ (3 bar) is added to 1 (5 mM), MPSO (25 mM) and NaOMe (50 mM) in methanol-d_3_ at 298 K. (b) Hydride region of a single scan ^1^H NMR spectra recorded with a 45° pulse a few minutes after *p*H_2_ (3 bar) is added to 1 (5 mM), MPSO (25 mM) and NaOMe (50 mM) in methanol-d_3_ at 298 K and shaken at 6.5 mT for 10 seconds. A thermally polarised spectrum recorded 30 seconds after this spectrum was recorded presented is shown above on the same vertical scale. The inset trace shows an expansion to illustrate the appearance of the polarised signals for 11 and 12. The singlet marked by the asterisk is tentatively assigned as an analogue of 11. (c) Hydride regions taken from ^1^H NMR spectra recorded a few days after H_2_ (3 bar) is added to 1 (5 mM), MPSO (25 mM) and KH (50 mM) with 18-crown-6 (25 mM) in toluene-d_8_ at 298 K.

Trihydride 8 contains two magnetically equivalent hydride ligands when *C*_s_-symmetric DMSO is used. However, when these experiments are repeated using asymmetric methylphenylsulfoxide (MPSO), analogues of 7 and 8 can be created wherein the hydride ligands are magnetically inequivalent. For example, reaction of 1, MPSO, NaOMe and H_2_ in either methanol-d_3_ or toluene-d_8_ yields equilibrium mixtures of [Ir(H)_4_(MPSO)(IMes)] (11) and [Ir(H)_3_(MPSO)_2_(IMes)] (12) (see ESI, Section S6† for details of their characterisation). When the hydride resonances for 12 are examined, two pairs of three distinct signals are now seen. This results from the fact 12 contains two chiral sulfoxides, and forms as two pairs of diastereomers, each with distinct resonances (Fig. 5a). A single hydride resonance was observed at *δ* = −8.44 for 11, which is analogous in position to that of 7.

Now, hyperpolarisation experiments in methanol-d_3_ immediately after the addition of *p*H_2_ to samples containing MPSO yield transiently enhanced signals for both diastereomers of 12, in analogy to those seen for 8 that were described earlier, although their relative intensity is now weaker. In addition, an enhanced singlet is seen for 11, alongside that of a further singlet at *δ* = −13.00 in methanol-d_3_ (Fig. 5b). These observations suggest that MPSO succeeds in breaking the symmetry of the precursors leading to 11. Unfortunately, the low proportion and stability of the species yielding these two singlets precluded their full NMR characterisation, however according to EXSY the two species are in exchange. We suggest therefore that they differ in respect to the location of the cation.

However, when these reactions are repeated in toluene-d_8_ using KH or KOMe as the base with 18-crown-6, the formation of asymmetric tetrahydride 11′ is achieved. It presents four inequivalent hydride ligands that resonate at *δ* = −11.75, −12.13, −14.21 and −14.95 (Fig. 5c, ESI S6† for characterisation). This inequivalence reflects the *cis* relationship between the sulfoxide and IMes ligands. The use of a crown ether to stabilise such facial arrangements has been demonstrated previously.^15^

### Formation and role of 6–9 during hyperpolarisation of pyruvate

**Fig. 6:**
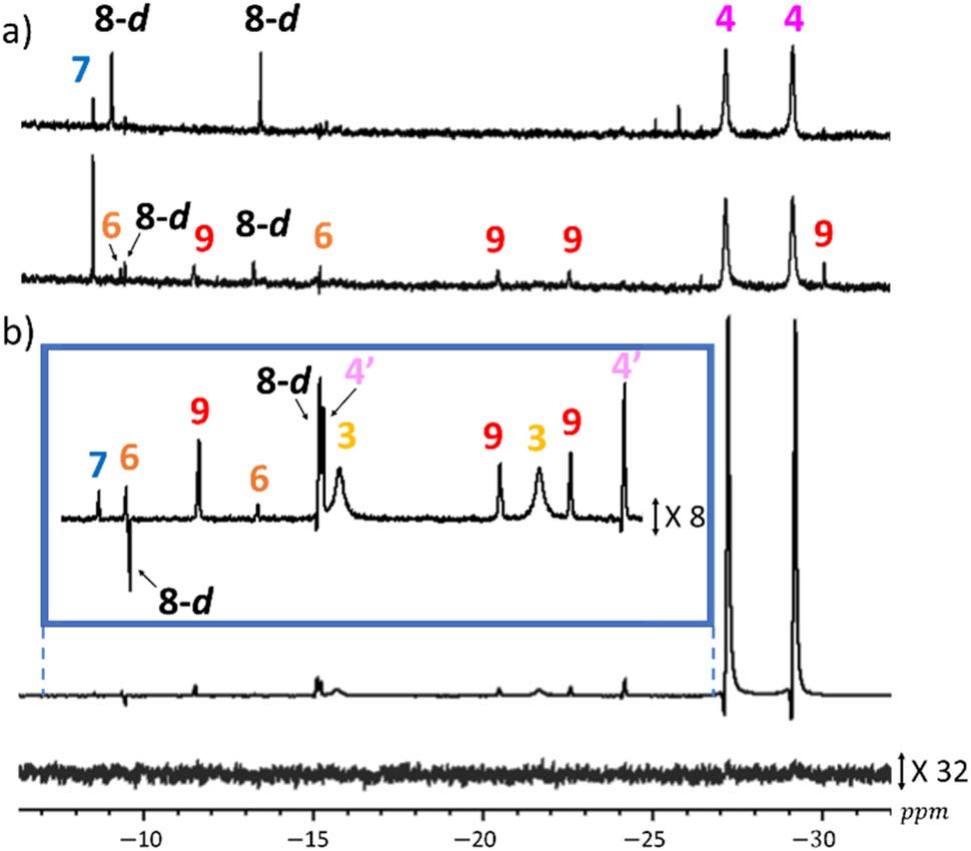
(a) ^1^H NMR spectra 1 min (upper) and 40 min (lower) after H_2_ (3 bar) addition to a solution of 1 (5 mM), DMSO (25 mM) and sodium pyruvate (30 mM) in methanol-d_4_ (0.6 mL) at 298 K. (b) Hyperpolarised single scan ^1^H NMR spectrum (and expansion in blue box with increased vertical scale) recorded using a 90° pulse using the solution for (a) but after shaking with 3 bar of *p*H_2_ for 10 seconds at 65 G, 15 minutes after the start of observations. The corresponding NMR spectrum at this time point, where the PHIP effect is absent and the vertical scale increased by a factor of 32 relative to (b) is shown below. For clarity, the peaks have been phased so the largest signals appear in absorption mode.

The formation of these *tri* and *tetra*hydride complexes in the analogous reactions with pyruvate was confirmed through analogous experiments where samples containing H_2_ (3 bar), 1 (5 mM), DMSO (25 mM) and sodium pyruvate (30 mM) were examined in methanol-d_4_ (0.6 mL) at 298 K. Now, signals for the hydride ligands of 3, and the two previously reported isomers of 4, are readily seen.^34,39^ However, when these NMR spectra are examined more closely, extra signals for 6, 7, 8, 8′, 8-d′, and 9 are seen, both in the corresponding thermally polarised and hyperpolarised ^1^H NMR spectra (Fig. 6). It is notable, that ^1^H NMR signals for these species are visible even though NaOMe is not specifically added to the samples. This is not surprising as sodium pyruvate equilibrates in methanol with NaOMe and pyruvic acid.

This hypothesis was confirmed by doping the solutions (1 (5 mM), DMSO (25 mM), and sodium pyruvate (30 mM) in methanol-d_4_ (0.6 mL)) with NaOMe (50 mM), and this did indeed favour the formation of 6–9. Interestingly, the expected signals for 4 are no longer seen. This agrees with the observation that when 4 is prepared *in situ*, by H_2_ addition to 1 (5 mM), DMSO (25 mM), and sodium pyruvate (30 mM) in methanol-d_4_ (0.6 mL), it rapidly converts into 7, 8 and 8-d′ once NaOMe (50 mM) is added.

In the context of pyruvate hyperpolarisation using SABRE, the presence of 6–9 will serve to hamper the key magnetisation transfer activity as their formation reduces the amount of active catalyst 4. However, as 6–9 appear to exhibit slow/no H_2_ exchange themselves they are unlikely to play a role in destroying the *p*H_2_ singlet order needed for SABRE. Hence, for those seeking to optimise pyruvate hyperpolarisation, the formation of 6–9 should simply be minimised, and thus sample pH controlled as it clearly is a key factor in the production of 7 and 8.

## Experimental

All NMR measurements were carried out on a 400 MHz Bruker Avance III spectrometer at 298 K unless otherwise stated. *Para*-hydrogen (*p*H_2_) was produced by passing hydrogen gas over a spin-exchange catalyst (Fe_2_O_3_) at 28 K and used for all hyperpolarisation experiments. This method produces constant *p*H_2_ with *ca.* 99% purity. ^1^H (400 MHz) and ^13^C (100.6 MHz) NMR spectra were recorded with an internal deuterium lock. Chemical shifts are quoted as parts per million and referenced to residual solvent. All starting compounds were purchased from Sigma Aldrich, Fluorochem, or Alfa-Aesar and used as supplied without further purification. 1 was synthesised according to a literature procedure.^54^

The shake & drop method was employed for recording hyperpolarised SABRE NMR spectra. Samples were prepared in a 5 mm NMR tube that was fitted with a J. Young's tap. NMR samples were subsequently degassed by three freeze–pump–thaw cycles using a Schlenk line before filling the tube with H_2_ or *p*H_2_ at 3 bar overpressure. Once filled with *p*H_2_, the tubes were shaken vigorously for 10 seconds at *ca.* 6.5 mT. Immediately after that, the NMR tubes were put inside the spectrometer for immediate NMR detection. Both hyperpolarised and thermally polarised spectra were recorded on the same sample using the same spectrometer settings.

## Conclusions

In this work novel trihydride [Ir(H)_3_(IMes)(sulfoxide)_2_] and tetrahydride [Ir(H)_4_(IMes)(sulfoxide)]M [M = Na and K] complexes result when [IrCl(COD)(IMes)] reacts with sulfoxide, base and H_2_. Mechanistic and kinetic ^1^H NMR studies have revealed that these complexes form in two separate pathways: either *via* [Ir(Cl)(H)_2_(IMes)(sulfoxide)_2_], or through replacement of the diene in [Ir(H)_3_(COD)(IMes)]. At long reaction times, the formation of dinculear complexes like [Ir_2_(Cl)(μ_2_-Cl)(H)_3_(μ_2_-H)(IMes)_2_(DMSO)_2_], and [Ir_2_(H)_4_(μ_2_-H)(μ_2_-OCD_3_)(DMSO)_2_(IMes)_2_] dominate the reaction products.

These reaction products have been characterised using a combination of advanced 2D NMR and *para*hydrogen hyperpolarised NMR. Notably, they can be detected with hyperpolarised NMR signals when *p*H_2_ is used as a reactant. Furthermore, the use of symmetry breaking methylphenylsulfoxide enables analogues of these complexes to be created where their hydride ligands become chemically distinct. This effect is a major benefit when analysing such systems by NMR spectroscopy and PHIP.

This study has also shown that these unusual products are present when related [Ir(H)_2_(κ^2^-pyruvate)(IMes)(sulfoxide)] and [IrCl(H)_2_(IMes)(sulfoxide)_2_] are used to catalytically transfer spin order from *p*H_2_ to pyruvate through SABRE. Significantly, the efficiency of pyruvate hyperpolarisation using SABRE has been dramatically increased from its initial demonstration in 2019 to the point where it has been used to produce probes for *in vivo* biomedical imaging studies in rodents with promise for clinical translation.^37^ Our results provide insight into the role these previously unknown species play during SABRE, and suggest their formation should be avoided, by varying sample pH in order to achieve the maximum active catalyst concentration.

## Data availability

The raw NMR data collected in this study is available from the University of York data repository.

## Author contributions

BJT – conceptualisation; investigation; validation; visualisation; writing – original draft, writing – review and editing. SBD – conceptualisation; investigation; validation; writing – review and editing; supervision, funding acquisition.

## Conflicts of interest

B. J. T. and S. B. D. (and others) are inventors on a patent application filed by the University of York related to this work (patent no. GB1818171.9).

## Supplementary Material

SC-OLF-D4SC06138A-s001

## References

[cit1] Babón J. C., Esteruelas M. A., López A. M. (2022). Chem. Soc. Rev..

[cit2] Sieffert N., Kendrick T., Tiana D., Morrison C. A. (2015). Dalton Trans..

[cit3] Van der Sluys L. S., Kubas G. J., Caulton K. G. (1991). Organometallics.

[cit4] Samouei H., Miloserdov F. M., Escudero-Adán E. C., Grushin V. V. (2014). Organometallics.

[cit5] Plois M., Hujo W., Grimme S., Schwickert C., Bill E., de Bruin B., Poettgen R., Wolf R. (2013). Angew. Chem., Int. Ed..

[cit6] Chan A. S. C., Shieh H.-S. (1985). Chem. Commun..

[cit7] Crabtree R. H., Hamilton D. G. (1986). J. Am. Chem. Soc..

[cit8] Guilera G., McGrady G. S., Steed J. W., Jones A. L. (2006). Organometallics.

[cit9] Gusev D. G., Lough A. J., Morris R. H. (1998). J. Am. Chem. Soc..

[cit10] Ott J., Venanzi L. M., Ghilardi C. A., Midollini S., Orlandini A. (1985). J. Organomet. Chem..

[cit11] Bakhmutov V. I., Bianchini C., Peruzzini M., Vizza F., Vorontsov E. V. (2000). Inorg. Chem..

[cit12] Göttker-Schnetmann I., White P. S., Brookhart M. (2004). Organometallics.

[cit13] Tanaka R., Yamashita M., Nozaki K. (2009). J. Am. Chem. Soc..

[cit14] Abdur-Rashid K., Gusev D. G., Landau S. E., Lough A. J., Morris R. H. (1998). J. Am. Chem. Soc..

[cit15] Landau S. E., Groh K. E., Lough A. J., Morris R. H. (2002). Inorg. Chem..

[cit16] Kisten P., Manoury E., Lledós A., Whitwood A. C., Lynam J. M., Slattery J. M., Duckett S. B., Poli R. (2023). Dalton Trans..

[cit17] Kubas G. J. (2014). J. Organomet. Chem..

[cit18] Kubas G. J. (2001). J. Organomet. Chem..

[cit19] Hövener J., Pravdivtsev A. N., Kidd B., Bowers C. R., Glöggler S., V Kovtunov K., Plaumann M., Katz-Brull R., Buckenmaier K., Jerschow A. (2018). Angew. Chem., Int. Ed..

[cit20] Rayner P. J., Duckett S. (2018). Angew. Chem., Int. Ed..

[cit21] Tickner B. J., V Zhivonitko V. (2022). Chem. Sci..

[cit22] Barskiy D. A., Knecht S., V Yurkovskaya A., Ivanov K. L. (2019). Prog. Nucl. Magn. Reson. Spectrosc..

[cit23] Adams R. W., Aguilar J. A., Atkinson K. D., Cowley M. J., Elliott P. I. P., Duckett S. B., Green G. G. R., Khazal I. G., López-Serrano J., Williamson D. C. (2009). Science (1979).

[cit24] Theis T., Truong M. L., Coffey A. M., V Shchepin R., Waddell K. W., Shi F., Goodson B. M., Warren W. S., Chekmenev E. Y. (2015). J. Am. Chem. Soc..

[cit25] Theis T., Truong M., Coffey A. M., Chekmenev E. Y., Warren W. S. (2014). J. Magn. Reson..

[cit26] Pravdivtsev A. N., Yurkovskaya A. V., Vieth H.-M., Ivanov K. L. (2015). J. Phys. Chem. B.

[cit27] Rayner P. J., Norcott P., Appleby K. M., Iali W., John R. O., Hart S. J., Whitwood A. C., Duckett S. B. (2018). Nat. Commun..

[cit28] Pham P., Hilty C. (2020). Chem. Commun..

[cit29] Cowley M. J., Adams R. W., Atkinson K. D., Cockett M. C. R., Duckett S. B., Green G. G. R., Lohman J. A. B., Kerssebaum R., Kilgour D., Mewis R. E. (2011). J. Am. Chem. Soc..

[cit30] Tickner B. J., Dennington M., Collins B. G., Gater C. A., Tanner T. F. N., Whitwood A. C., Rayner P. J., Watts D. P., Duckett S. B. (2024). ACS Cat..

[cit31] Tickner B. J., John R. O., Roy S. S., Hart S. J., Whitwood A. C., Duckett S. B. (2019). Chem. Sci..

[cit32] Kubas G. J. (1988). Acc. Chem. Res..

[cit33] Kubas G. J. (2014). J. Organomet. Chem..

[cit34] Iali W., Roy S. S., Tickner B. J., Ahwal F., Kennerley A. J., Duckett S. B. (2019). Angew. Chem., Int. Ed. Engl..

[cit35] Tickner B. J., Semenova O., Iali W., Rayner P. J., Whitwood A. C., Duckett S. B. (2020). Cat. Sci. Technol..

[cit36] TomHon P., Abdulmojeed M., Adelabu I., Nantogma S., Kabir M. S. H., Lehmkuhl S., Chekmenev E. Y., Theis T. (2022). J. Am. Chem. Soc..

[cit37] Schmidt A. B., de Maissin H., Adelabu I., Nantogma S., Ettedgui J., TomHon P., Goodson B. M., Theis T., Chekmenev E. Y. (2022). ACS Sens..

[cit38] de Maissin H., Groß P. R., Mohiuddin O., Weigt M., Nagel L., Herzog M., Wang Z., Willing R., Reichardt W., Pichotka M. (2023). Angew. Chem., Int. Ed..

[cit39] Tickner B. J., Lewis J. S., John R. O., Whitwood A. C., Duckett S. B. (2019). Dalton Trans..

[cit40] Assaf C. D., Gui X., Auer A. A., Duckett S. B., Hövener J.-B., Pravdivtsev A. N. (2024). J. Phys. Chem. Lett..

[cit41] TomHon P., Abdulmojeed M., Adelabu I., Nantogma S., Kabir M. S. H., Lehmkuhl S., Chekmenev E. Y., Theis T. (2021). J. Am. Chem. Soc..

[cit42] Tickner B. J., Ahwal F., Whitwood A. C., Duckett S. B. (2021). ChemPhysChem.

[cit43] Tickner B. J., Parker R. R., Whitwood A. C., Duckett S. B. (2019). Organometallics.

[cit44] Tickner B. J., Whitwood A. C., Condon C., Platas-Iglesias C., Duckett S. B. (2024). Eur. J. Inorg. Chem..

[cit45] Giernoth R., Huebler P., Bargon J. (1998). Angew. Chem., Int. Ed..

[cit46] Bargon J. , The Handbook of Homogeneous Hydrogenation, 2006, pp. 313–358

[cit47] Ahlquist M., Gustafsson M., Karlsson M., Thaning M., Axelsson O., Wendt O. F. (2007). Inorg. Chim. Acta.

[cit48] Godard C., Duckett S. B., Polas S., Tooze R., Whitwood A. C. (2005). J. Am. Chem. Soc..

[cit49] Duckett S. B., Newell C. L., Eisenberg R. (1994). J. Am. Chem. Soc..

[cit50] V Kireev N., Kiryutin A. S., Pavlov A. A., Yurkovskaya A. V., Musina E. I., Karasik A. A., Shubina E. S., Ivanov K. L., V Belkova N. (2021). Eur. J. Inorg. Chem..

[cit51] Blazina D., Duckett S. B., Dyson P. J., Lohman J. A. B. (2003). Chem.–Eur. J..

[cit52] Duckett S. B., Wood N. J. (2008). Coord. Chem. Rev..

[cit53] Gobetto R., Milone L., Reineri F., Salassa L., Viale A., Rosenberg E. (2002). Organometallics.

[cit54] Vazquez-Serrano L. D., Owens B. T., Buriak J. M. (2006). Inorg. Chim. Acta.

