# Supplementary material for: Iridium trihydride and tetrahydride complexes and their role in catalytic polarisation transfer from parahydrogen to pyruvate
Source: Chem Sci. 2024 Dec 12;16(3):1396–404. doi: 10.1039/d4sc06138a (PMC11654498; doi:10.1039/d4sc06138a)
Supplement: SC-016-D4SC06138A-s001 [file SC-016-D4SC06138A-s001.pdf]

Supplementary Information (SI) for Chemical Science.
This journal is © The Royal Society of Chemistry 2024

Supporting Information

Iridium trihydride and tetrahydride complexes and their role in catalytic polarisation transfer from *para*hydrogen to pyruvate

Ben. J. Tickner[a] and Simon B. Duckett*[a]

[a.] *Centre for Hyperpolarisation in Magnetic Resonance, Department of Chemistry, University of York, Heslington, YO10 5NY, United Kingdom*
**Corresponding Author email: simon.duckett@york.ac.uk*

Table of Contents

S1 2D NMR characterisation of 3 and 5

S2 Characterisation of 7 and 8
- S2.1 NMR
- S2.2 HRMS

S3 NMR Characterisation of 9

S4 Formation of 7 and 8 from 3

S5 Hyperpolarisation of 8

S6 NMR Characterisation of 11 and 12

S7 2D NMR pulse sequences
- S7.1 HMQC
- S7.2 HSQC
- S7.3 NOESY
- S7.4 COSY
- S7.5 Selective NOESY
- S7.6 Selective COSY

S8 References

S1 2D NMR Characterisation of 3 and 5

A solution of **1** (5 mM) and DMSO (25 mM) in methanol-*d*$_3$ was reacted with H_2 (3 bar), This led to the formation of a mixture containing **3**, **5** and **5'**. A typical ^{1}H NMR spectrum of this mixture is shown in Figure S1.

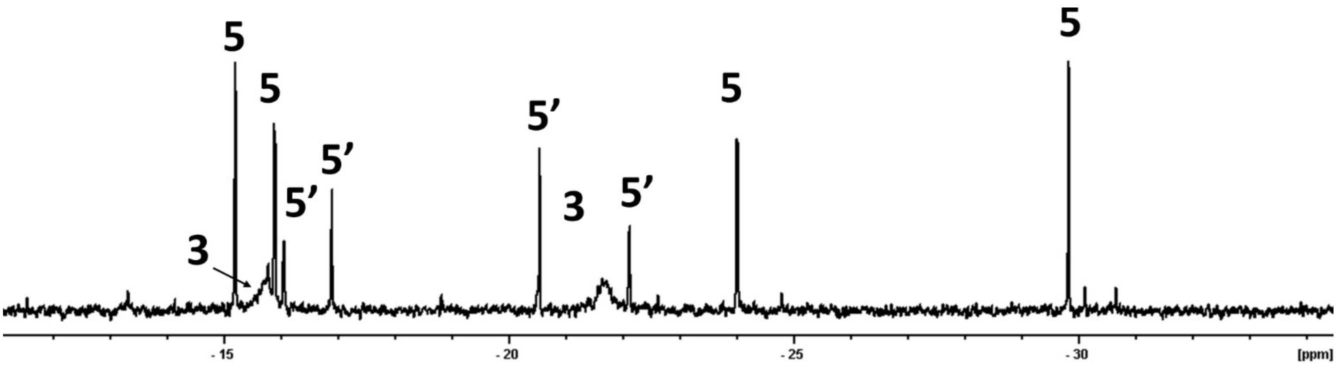

***Figure S1:** ^{1}H NMR spectrum at 298 K of a mixture of 3, 5, and 5' in methanol-d$_3$.*

These species were characterised using 2D NMR spectroscopy at 253 K. The structure of **3** is shown in Figure S2 and its NMR characterisation data given in Table S1. These data are consistent with that previously reported for **3**.[1] **3** is highly reactive, containing a very labile sulfoxide, which results in the hydride ligand signals showing substantial broadening at 298 K. In CD_3OD this results in their rapid deuteration at 298 K, with the concomitant formation of $IrCl(D)_2(DMSO)_2$(IMes) and no hydride ligand signals. In CD_3OH, these signals remain visible, albeit they are broadened substantially. Under conditions where the excess of DMSO is low, they again become barely visible, with the corresponding signal for H_2 showing substantial broadening. Cooling to 253 K returns sharper hydride ligand signals, for which the H-H spitting of 6 Hz can be resolved.

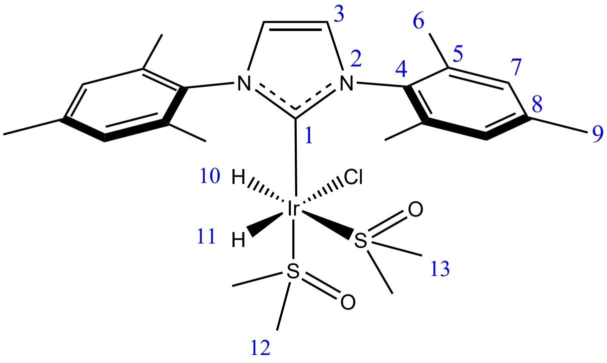

***Figure S2:** Structure of 3*

***Table S1:** NMR resonance for 3 in methanol-d$_3$ at 253 K. The resonance labels correspond to those shown in Figure S2.*

Resonance Number	^{1}H / ppm	^{13}C / ppm	^{15}N / ppm
1		157.0	
2			197.99
3	7.22	122.87	
4		139.33	
5		135.74	
6	2.18	17.81, 18.20	
7	7.00	128.56	
8		137.70	
9	2.37	19.96	
10	− 15.53 (*d*, 6 Hz)		
11	− 21.57 (*d*, 6 Hz)		
12	3.18, 3.26	43.58, 57.52	
13	2.81, 3.11	54.47, 39.73	

The structure of **5** is shown in Figure S3 and its NMR resonances are shown in Table S2. The structure is confirmed from control experiments involving [IrBr(COD)(IMes)] precursors as the hydride ligands of **5** at 298 K appear at δ − 15.79, − 16.06, − 24.80, and − 30.66 when formed from **1**, but shift to δ − 15.64, − 15.88, − 22.63, and − 30.10 when formed from [IrBr(COD)(IMes)]. The significant shift (2.2 ppm) of H_{11} is consistent with its assignment *trans* to bridging halide. The remaining hydride ligands, H_{10} and H_{13} only shift by < 0.2 ppm consistent with their assignment *cis* to halide. The shift differences for H_{14} are 0.6 ppm at 298 K, but only 0.25 Hz at 253 K, and thereby consistent with a *cis* relationship to two halides. The hydride ligand signals for **5** at 253

K appear at δ −15.20, −15.80, −23.96, and −29.70. They are all interconnected by COSY (Figure S4) and nOe cross peaks (Figure S5). All four signals are connected by COSY, with the exceptions of the resonance pairs at δ −15.20, −15.80 and δ −23.96, and −29.70. The signal at δ −29.70 shows nOe to the signal at δ −15.20, and the pair at δ −15.80 and −23.96 are also connected by nOe. NOESY, COSY, and HMQC/HSQC experiments collectively allowed the hydride ligand at δ −15.20 to be assigned as a bridging hydride ligand, it is connected to DMSO ligands on two different metal centres by a combination of COSY and NOE. The hydrides at δ −15.80, −23.96 show a mutual 9 Hz coupling and both couple by NOE and COSY to the same DMSO and IMes ligand. They are placed *trans* to bridging hydride and chloride respectively. The remaining hydride ligand at δ −29.70 is placed *trans* to the bridging chloride on the second iridium centre, and it also shows a COSY connection to the same DMSO which is linked to the bridging hydride. The remaining resonances are also located through similar NOESY, COSY, and HMQC/HSQC experiments which further confirm the structure.

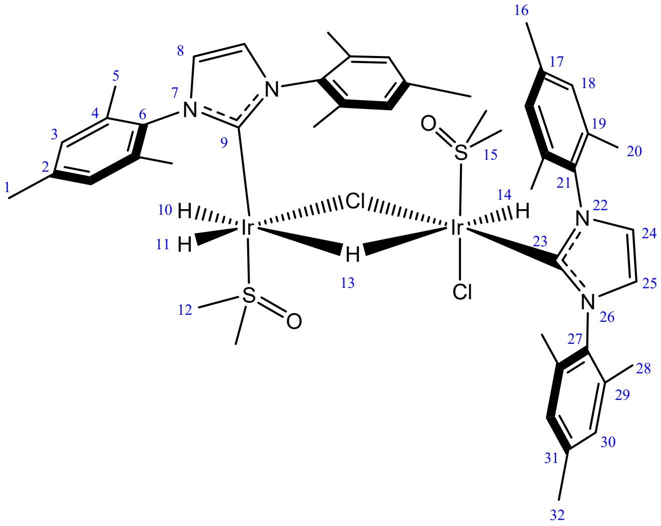

Figure S3: Structure of 5

Table S2: NMR resonances of 5 in methanol-d_3 at 253 K. The resonance labels correspond to those shown in Figure S3.

	Starting from [IrCl(COD)(IMes)]			Starting from [IrBr(COD)(IMes)]		
Resonance Number	***^{1}H / ppm***	***^{13}C / ppm***	***^{15}N / ppm***	***^{1}H / ppm***	***^{13}C / ppm***	***^{15}N / ppm***
**1**	2.33	19.81		2.32	19.85	
**2**		138.68			-	
**3**	6.90, 7.03	128.78, 128.53		6.88, 7.03	128.85, 128.61	
**4**		135.00			135.23, 135.70	
**5**	1.93, 2.03	17.56, 17.48		1.93, 2.05	17.58, 17.62	
**6**		137.66			137.91	
**7**			197.06			197.66
**8**	7.24	122.86		7.21	123.40	|
**9**		158.36			-	
**10**	−15.80 (*d*, $^{2}J_{HH}$ = 9 Hz)			−15.83 (*d*, $^{2}J_{HH}$ = 8 Hz)		
**11**	−23.96 (*d*, $^{2}J_{HH}$ = 9 Hz)			−22.56 (*d*, $^{2}J_{HH}$ = 8 Hz)		
**12**	2.37, 2.98	49.66, 49.21		2.31, 3.03	50.29, 49.91	
**13**	−15.20 (*br*)			−15.63 (*br*)		
**14**	−29.70 (*d*, $^{2}J_{HH}$ = 3 Hz)			−29.95 (*d*, $^{2}J_{HH}$ = 3 Hz)		
**15**	2.78, 3.02	54.78, 48.51		2.65, 3.05	54.58, 48.62	
**16**	2.47	19.91		2.51	19.99	
**17**		139.25			139.24	
**18**	7.13, 7.18	129.46, 129.28		7.18, 7.19	129.30, 129.59	
**19**		136.46, 135.96			136.54, 136.10	
**20**	2.08, 2.24	18.23, 17.53		2.11, 2.24	18.76, 17.64	
**21**		137.41			137.27	
**22**			197.18			197.55
**23**		148.32			-	
**24**	7.46	123.66		7.46	123.47	
**25**	7.16	125.42		7.17	125.53	
**26**			198.16			197.87
**27**		135.27			135.14	
**28**	2.12, 2.14	17.43, 17.72		2.09, 2.19	17.53, 18.45	
**29**		137.88, 135.92			138.28, 135.66	
**30**	6.87, 6.93	128.19, 128.09		6.86, 6.93	128.22, 127.94	
**31**		138.59			138.59	
**32**	2.14	17.72		2.27	19.65	

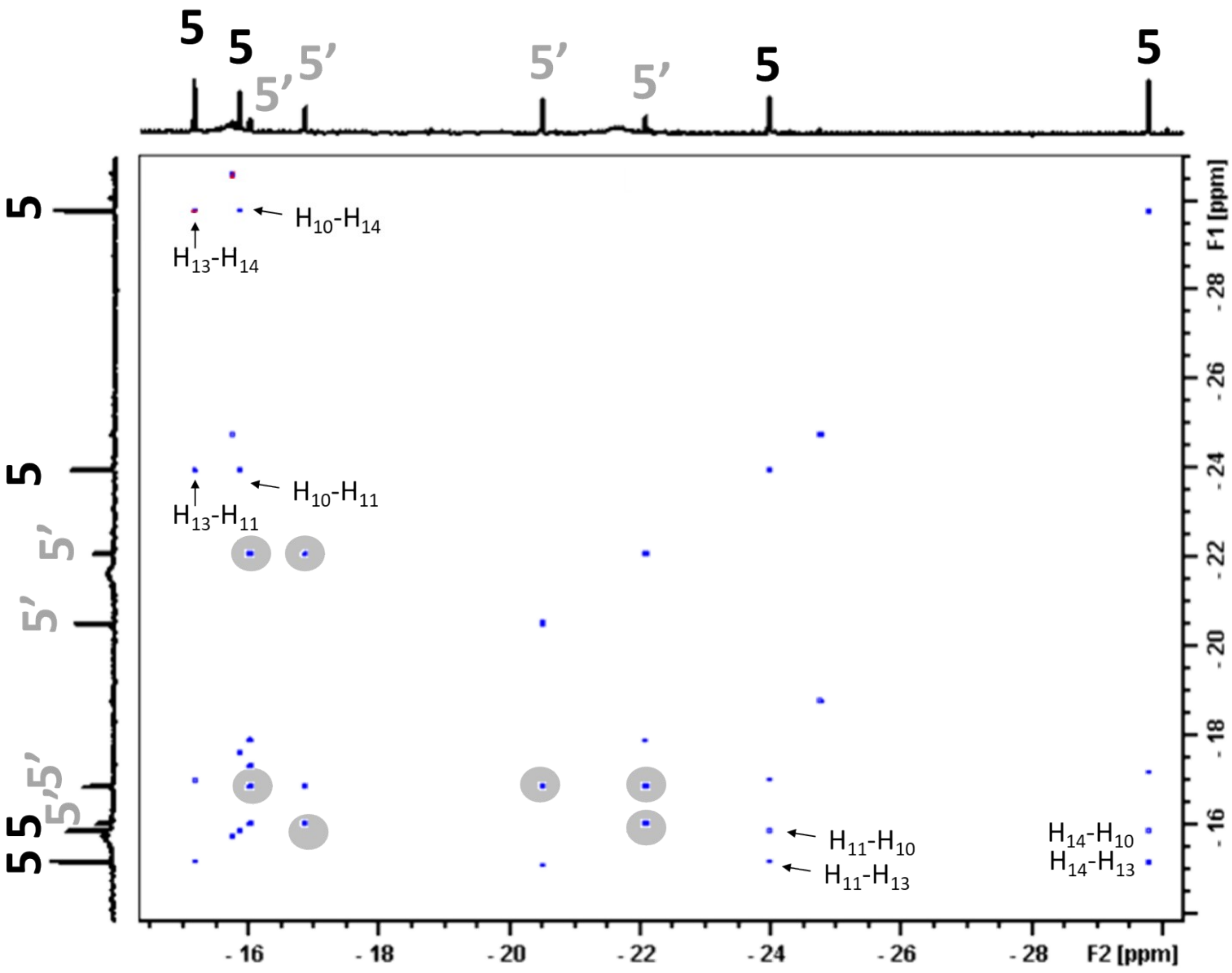

Figure S4: ^{1}H COSY in methanol-d_3 at 245 K for a solution of 3,5 and 5'

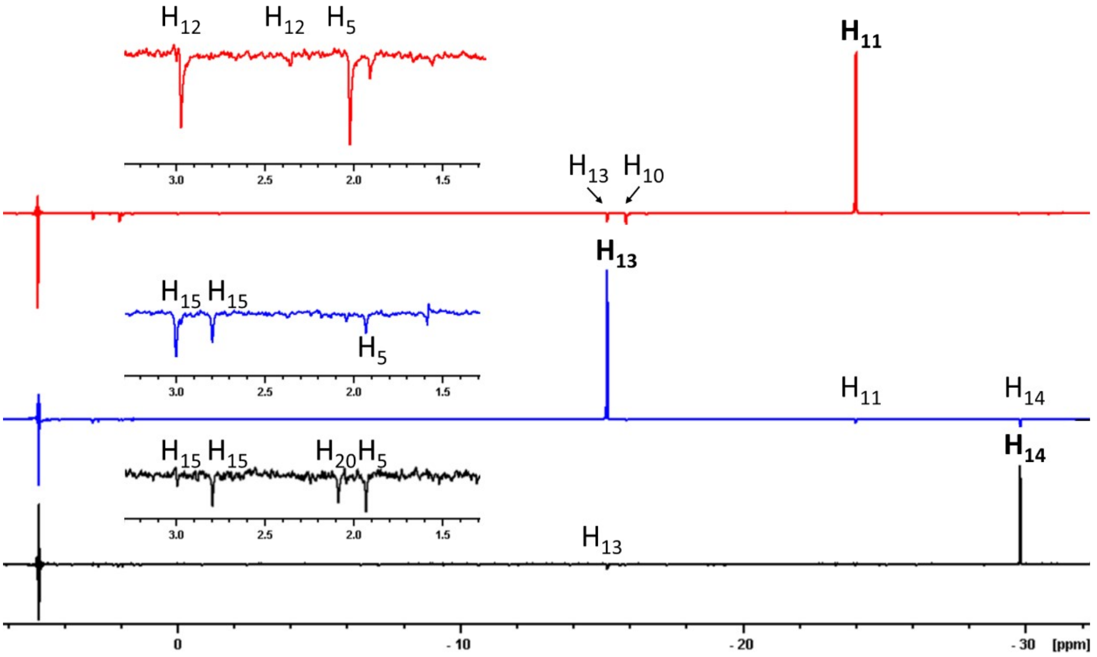

Figure S5: ^{1}H selective NOESY in methanol-d_3 at 245 K for a solution of 3,5 and 5'. The sites of 5 denoted in bold are selectively excited. The insets show a zoom in of the aliphatic region.

In these experiments a second set of signals are observed for **5'**, which is assigned as a minor isomer of **5**. It could not be fully characterised by 2D NMR due to its low concentration, but its hydride ligand signals appear at δ −16.90, −18.82, −20.55, −22.11 at 298 K. The signals at δ −16.90, and δ −22.11 are mutually coupled (J = 7 Hz) and exhibit comparatively small shifts (1-2 ppm) compared to the analogous sites in **5**. Therefore, the arrangement of ligands around one metal centre in **5'** is expected to be the same as in **5**. Notably, the signal at δ −20.55 in **5'** is significantly shifted (9 ppm) from its counterpart in **5**, which is consistent with a now *trans* hydride-DMSO relationship around the second metal centre in **5'**, compared to *cis* in **5**. The bridging hydride ligand signal at δ −18.82 in **5'** is now *trans* to halide and it exhibits a 1.3 ppm shift when formed from [IrBr(COD)(IMes)], which was not observed for the analogous site in **5**. Therefore, the bridging hydride is now *trans* to Cl in **5'**, compared to *cis* in **5**. At 253 K the hydride ligand signals of **5'** appear at δ −16.05, −16.88, −20.54, −22.21. The structure of **5'** is shown in Figure S6.

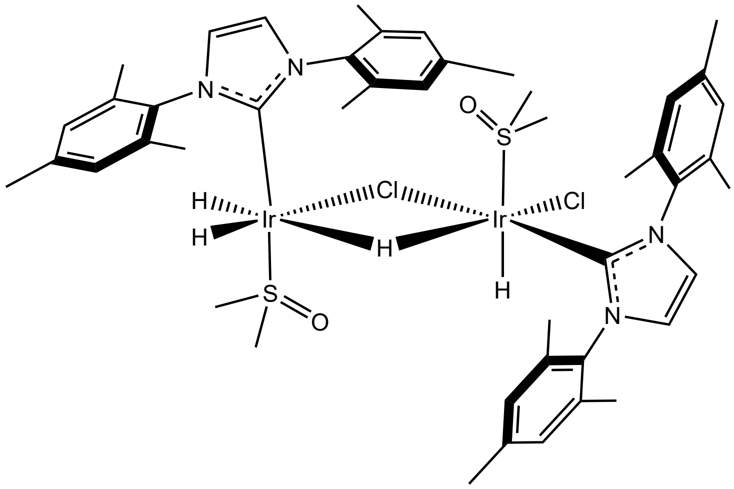

Figure S6: Structure of 5'

S2 Characterisation of 7 and 8

S2.1 NMR

A solution of **1** (5 mM), DMSO (25 mM) and NaOMe (50 mM) in methanol-d_3 or DCM-d_2 was reacted with H_2 (3 bar) for *ca* 30 mins at room temperature to form a mixture of **7** and **8**. These species were characterised using 2D NMR spectroscopy.

The structure of **7** is shown in Figure S7 and its NMR resonances are given in Table S3. The structure of **8** is shown in Figure S8, and its NMR resonances are given in Table S4.

The T_1 for the hydride resonance of **7** in methanol-d_3 was determined at 9.4 T and 234 K as 746 ms (at δ −8.59). This compares with the T_1 values for the two hydride ligand signals of **8** at δ −9.54 and δ −15.06 of 577 ms and 413 ms respectively. These data confirm that the hydride ligands in **7** are classical in nature, as a normal T_1 value is returned. Furthermore, the appearance of its hydride ligand signal remains as a sharp singlet even at 222 K, indicating the presence of no symmetry breaking dynamic effects such as those which would be associated with a dihydride-dihydrogen isomer.

NOESY (Figure S9) and COSY (Figure S10) spectra do not reveal connections from the hydride signal of **7** to any other hydride signals. An NOE connection is observed to an IMes signal at δ 2.01 and a DMSO signal at δ 3.22. The hydride and DMSO signal also show a COSY connection. Integration of the hydride ^{1}H NMR signals and the IMes signal at δ 2.01, which does not overlap with anything else, shows that the hydride ligand and IMes are in a 4:1 ratio. The remaining ^{1}H and ^{13}C resonances were located using ^{1}H-^{13}C HMQC/HSQC (Figure S11) and ^{1}H COSY (Figure S12) experiments. A *cis* hydride-IMes relationship was further confirmed in a control experiment using ^{13}C-IMes labelled at the carbene carbon which revealed the hydride ligand signal appears as a doublet with $^2J_{HC}$ = 4 Hz (Figure S13). Both **7** and **8** were confirmed to be monomeric using DOSY as their diffusion coefficients were comparable to **3**.

Note that characterisation data for **6** has been presented previously.[2]

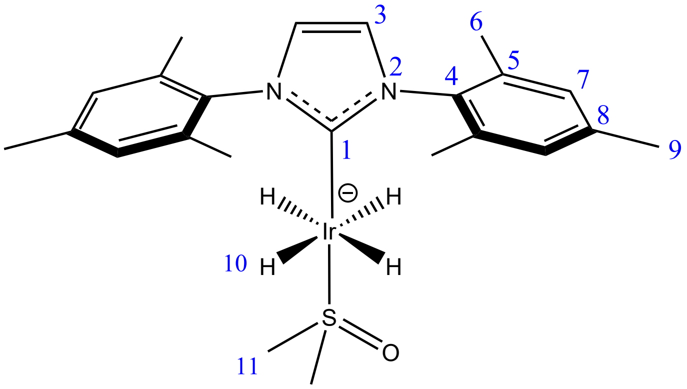

Figure S7: Structure of 7

Table S3: NMR resonances of 7. The resonance labels correspond to those shown in Figure S7.

	Methanol-d_3 at 253 K			***DCM-d_2 at 298 K***	
**Resonance Number**	**^{1}H / ppm**	**^{13}C / ppm**	**^{15}N / ppm**	**^{1}H / ppm**	**^{13}C / ppm**
**1**		150.50			150.97
**2**			194.42		
**3**	7.09 (2H)	120.87		6.94	122.96
**4**		138.24			-
**5**		135.02			135.39
**6**	2.01 (12H)	17.53		2.03	18.46
**7**	7.05 (4H)	128.76		7.08	128.72
**8**		138.43			138.33
**9**	2.39	19.97		2.43	20.98
**10**	− 8.58 (4H)			− 8.68	
**11**	3.22	57.98		3.18	59.71

The hydride ligand NMR signals of **8** at *δ* − 9.59 and − 15.12 belong in the same complex as they are coupled in a COSY spectrum. They appear in an integral ratio of *ca* 2:1 and as the chemical shifts are consistent with environments *trans* to hydride and sulfoxide respectively it is likely they correspond to a species that contains a DMSO ligand in the same plane as three hydrides. A NOE cross peak is observed from both hydrides to an IMes resonances at *δ* 2.15, confirming the IMes ligand is *cis* to all hydrides. The hydride at *δ* − 9.59 is confirmed *cis* to a DMSO *via* an NOE connection to *δ* 3.16, and that at *δ* − 15.12 *cis* to a DMSO *via* an NOE connection to *δ* 3.35. The DMSO ligand *cis* to both hydrides is confirmed at *δ* 3.35 as this resonance, which overlaps with the methanol solvent peak, shows a strong COSY cross peak to both hydrides. This signal is shown to couple to a ^{13}C resonance at *δ* 59.84 and is therefore consistent with DMSO (the methanol solvent signal couples to the methanol carbon at *δ* 48.62). The remaining ^{1}H and ^{13}C resonances were located using ^{1}H-^{13}C HMQC/HSQC and ^{1}H COSY experiments.

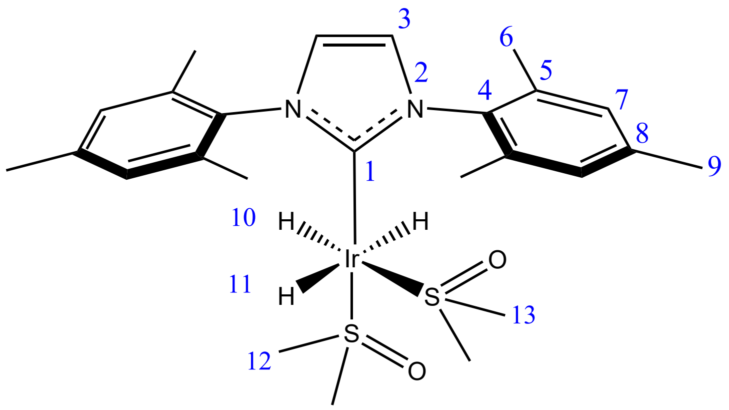

Figure S8: Structure of 8

Table S4: NMR resonances of 8. The resonance labels correspond to those shown in Figure S8.

	Methanol-d_3 at 253 K			***DCM-d_2 at 298 K***	
**Resonance Number**	**^{1}H / ppm**	**^{13}C / ppm**	**^{15}N / ppm**	**^{1}H / ppm**	**^{13}C / ppm**
**1**		157.00			157.49
**2**			197.99		
**3**	7.16	121.70		6.92	121.34
**4**		139.33			139.57
**5**		135.74			136.21
**6**	2.15	18.28		2.15	18.82
**7**	7.00	128.30		7.01	128.44
**8**		137.70			137.71
**9**	2.37	19.95		2.41	20.98
**10**	− 15.12 (*t*, 5 Hz)			− 15.41	
**11**	− 9.59 (*d*, 5 Hz)			− 9.53	
**12**	3.35	61.84		3.34	61.58
**13**	3.16	55.16		3.14	55.09

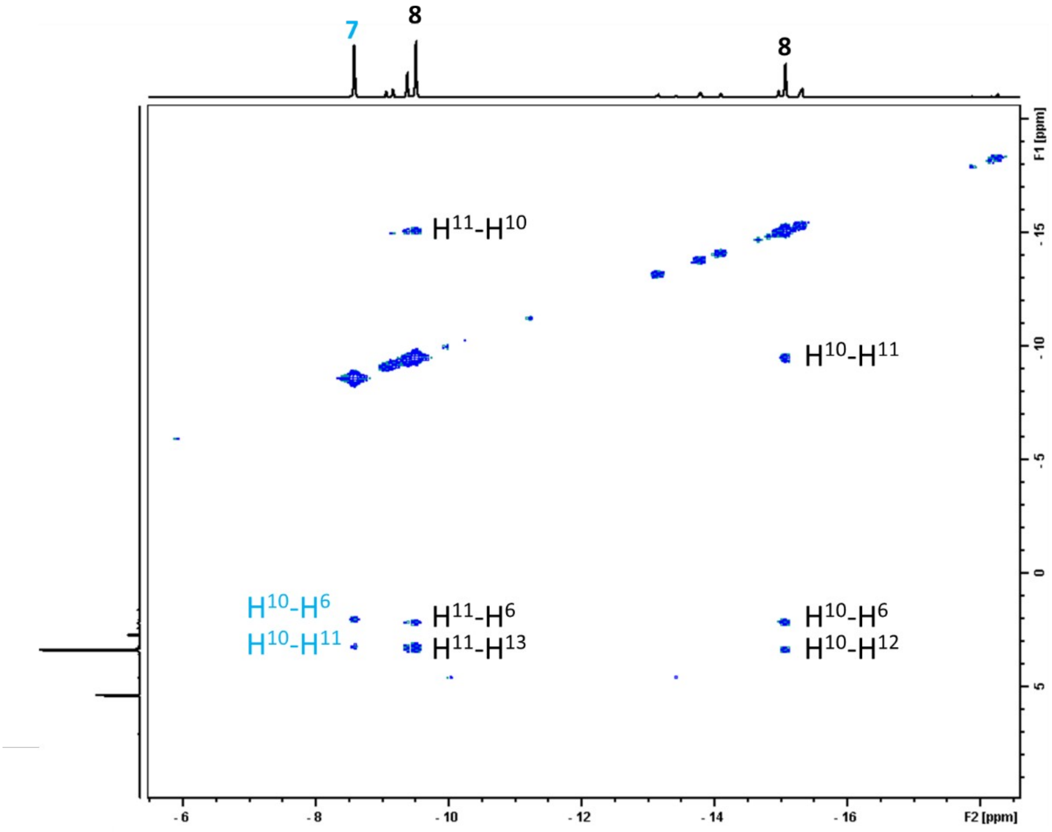

Figure S9: ${}^{1}H$ NOESY spectrum recorded in methanol-d_4 at 245 K.

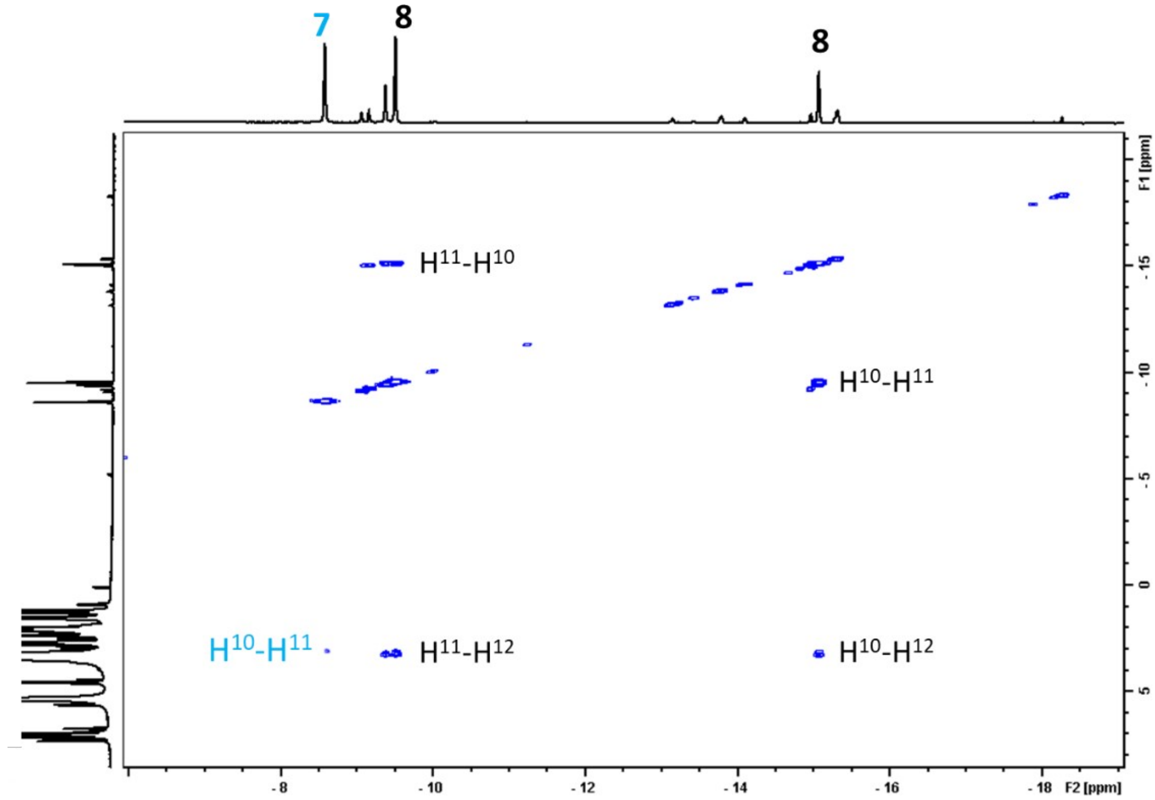

Figure S10: ${}^{1}H$ COSY spectrum recorded in methanol-d_4 at 245 K.

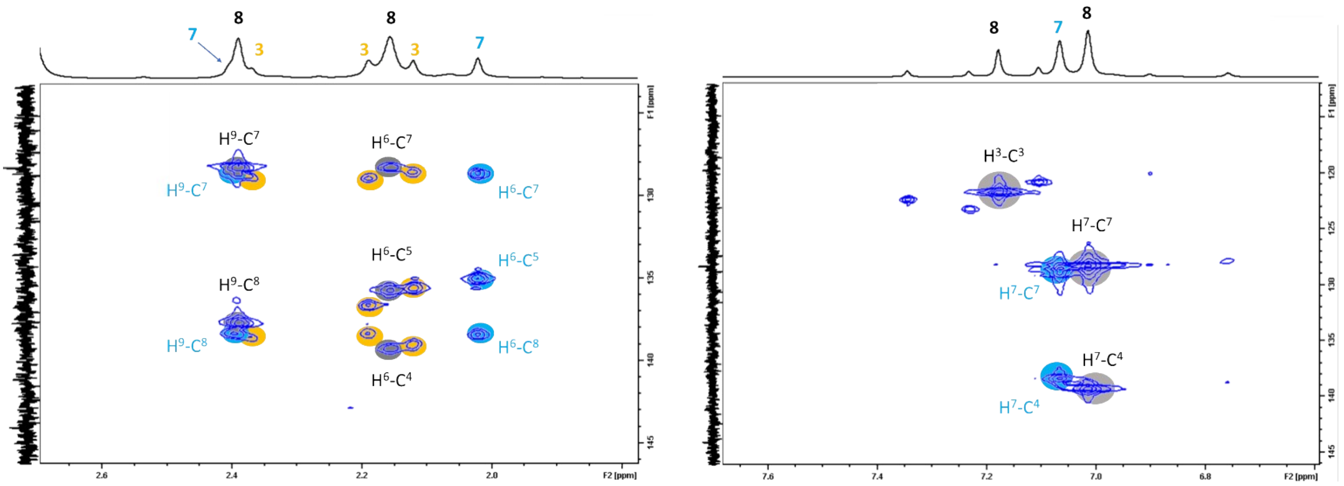

***Figure S11:** *^{1}H-^{13}C HMQC spectra optimised for a 12 Hz ^{1}H-^{13}C J coupling recorded in methanol-d$_4$ at 245 K. Note that 3 is also present in this mixture, its signals are highlighted in green and has been reported elsewhere.*[2]

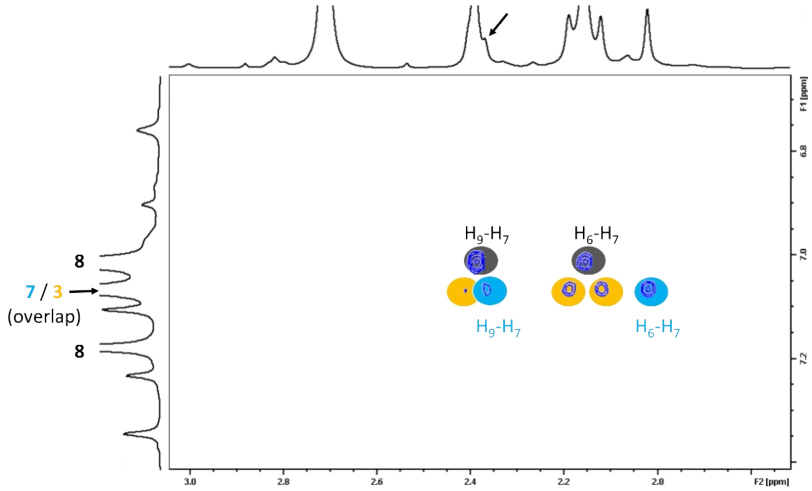

***Figure S12:** *^{1}H COSY spectrum recorded in methanol-d$_4$ at 245 K. Note that 3 is also present in this mixture, its signals are highlighted in green and have been reported elsewhere.*[2]

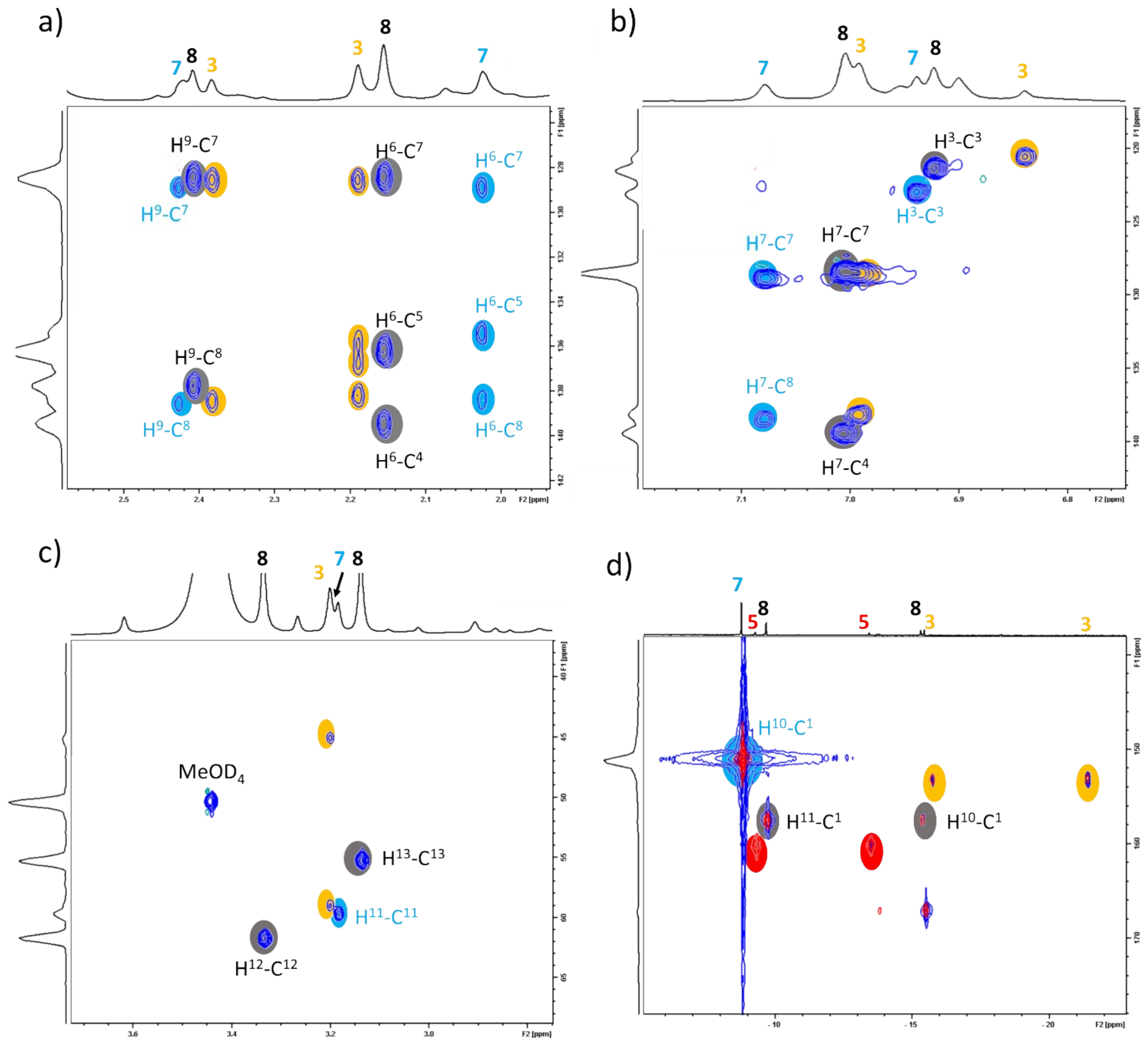

Figure S13: ^{1}H-^{13}C HSQC spectra optimised with a 12 Hz ^{1}H-^{13}C J coupling recorded in DCM-d_2 at 298 K. Note that 3 and 5 also present in this mixture, their signals are highlighted in green and red respectively and have been reported elsewhere.[1,2] *Note that in d) ^{13}C-IMes was used and spectra were recorded optimised with 4 Hz (blue) and 20 Hz (red) ^{1}H-^{13}C J couplings.*

A minor isomer of **8**, **8'** also forms. At 298 K, this species exists in a 13% proportion. Its hydride resonances were found to couple to DMSO resonances at δ 2.82 and 3.17 in COSY spectra. Both hydrides were close in space to an IMes resonance at δ 2.15, which overlaps with the signal of **8**. This signal overlap and the low abundance of this isomer made full NMR characterisation challenging. The structure of **8'** is shown in Figure S14 and its NMR partial NMR resonances given in Table S5.

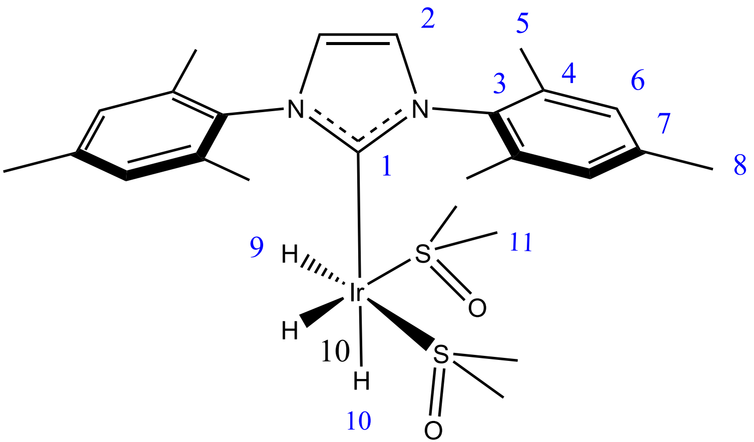

Figure S14: Structure of 8'

Table S5: NMR resonances of 8' in methanol-d_3 at 253 K. The resonance labels correspond to those shown in Figure S14.

Resonance Number	^{1}H / ppm	^{13}C / ppm
1		165.52
2	7.23	123.13
3-8	overlap with **8**	overlap with **8**
9	−15.48 (*d*, 5 Hz)	
10	−13.85 (*d*, 5 Hz)	
11	2.82, 3.17	55.17, 55.76

S2.2 HRMS

Electrospray ionization high resolution mass spectrometry was performed on solutions containing **3, 7** and **8** in methanol prepared as previously described, but in *protio* methanol solvent. These revealed peaks which correspond to fragments formed by DMSO loss from **3** and **8**. Calculated $C_{22}H_{32}Cl_2IrN_2O$ [Ir + IMes + Cl + H_2 + Cl^- + $2H^+$ + CH_3OH] = [**3** – 2DMSO + Cl^- + $2H^+$ + CH_3OH] m/z 603.1521, found 603.1966 (Figure S15). Calculated [Ir + IMes + H_3 + $2Cl^-$ + $3H^+$] = [**8** – 2DMSO + $2Cl^-$ + $3H^+$] $C_{21}H_{30}Cl_2IrN_2$ m/z 573.1415, found 573.1191 (Figure S15). Note that both peaks have an isotope pattern indicative of Cl_2 species.

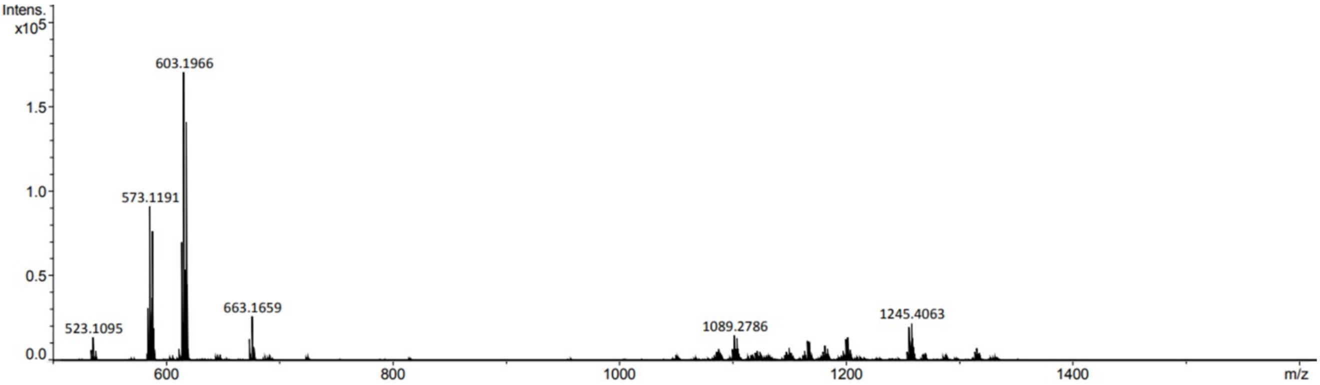

Figure S15: High resolution ESI mass spectrum of a mixture containing 3 and 8.

S3 NMR Characterisation of 9

1 (5 mM), DMSO (25 mM) and NaOMe (50 mM) in methanol-d_3 react with H_2 (3 bar) to form **7** and **8** as described earlier. **9** is also formed, but its proportion can be maximised if the reaction is left overnight at room temperature before it is characterised by cooling to 243 K. **9** has hydrides at δ −11.80, −13.21, −20.30, −22.48, −29.77 at 253 K. Its structure is given in Figure S16 and its NMR resonances in Table S6. When ^{13}C-IMes is used as the precursor the hydrides at δ −11.80 and −13.21 appear as doublets with a $^2J_{HC}$ coupling of 20 Hz and 33 Hz respectively, indicating they are both *trans* to different IMes ligands, and supported by their chemical shift. The hydrides at δ −11.80, −13.21, −29.77 are connected (NOESY, Figure S17 and COSY, Figure S18). The hydride at δ −13.21 is located as a bridging hydride as while it is *trans* to an IMes it also shows an nOe to an IMes resonance at δ 2.00. Furthermore, this bridging hydride is also connected by COSY to hydrides at δ −20.30, −22.48, which show mutual COSY and NOESY connections. Notably, when **9** is formed from [IrBr(COD)(IMes)] none of its hydride resonances shift, therefore the structure is not expected to contain halide and accordingly a methoxide is placed as the second bridging ligand which is *trans* to the hydrides at δ−22.48, −29.77.

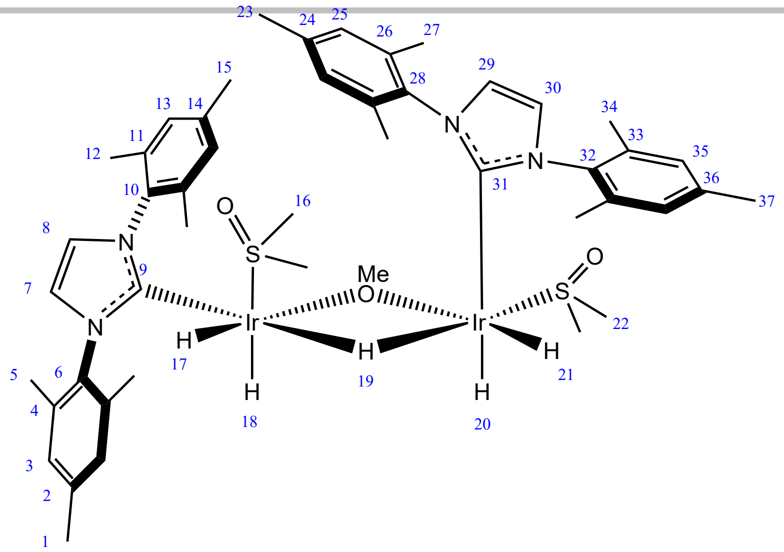

Figure S16: Structure of 9

Table S6: NMR resonances of 9 in methanol-d$_3$ at 243 K. The resonance labels correspond to those shown in Figure S16.

Resonance Number	^{1}H / ppm	^{13}C / ppm
**1**	2.45 (overlap with H_{15})	19.86 (overlap with C_{15})
**2**		139.38
**3**	7.12	129.47
**4**		-
**5**	2.14	17.73
**6**		137.14
**7**	7.35	123.59
**8**	7.05 (overlap with H_{13})	125.53
**9**		-
**10**		137.34
**11**		134.86
**12**	1.95, 2.01	17.58, 18.15
**13**	7.05 (overlap with H_8), 7.14	129.05, 128.40
**14**		138.40
**15**	2.45 (overlap with H_1)	19.86 (overlap with C_{15})
**16/22**	2.47, 2.92<br>2.87, 3.10	50.99, 51.90<br>48.66, 47.10
**17**	−22.48 (*d*, *J* = 8 Hz)	
**18**	−20.30	
**19**	−13.21	
**20**	−11.80	
**21**	−29.77 (*t*, *J* = 4 Hz)	
**23**	-	-
**24**		-
**25**	6.87	128.37
**26**		134.42
**27**	2.00	18.04
**28**		136.11
**29**	7.37	122.28
**30**	7.34	122.94
**31**		155.57
**32**		137.16
**33**		135.99
**34**	2.11	19.25
**35**	7.11	129.36

**36**		-
**37**	2.44	19.86

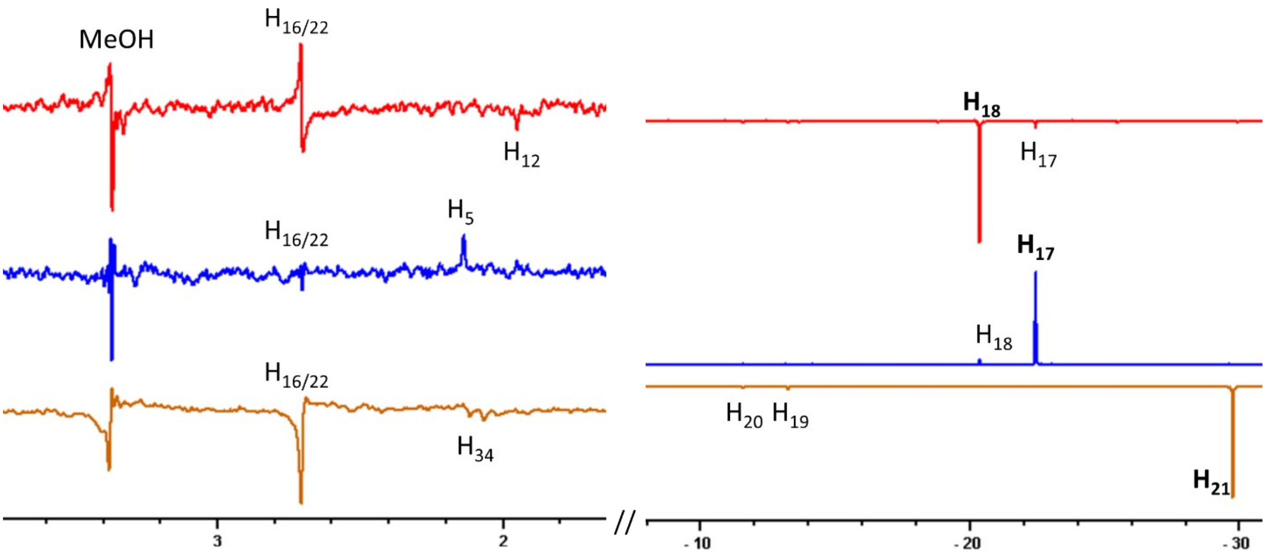

Figure S17: ^{1}H selective NOESY in methanol-d_3 at 245 K for a solution of 9. The sites of 9 denoted in bold are selectively excited.

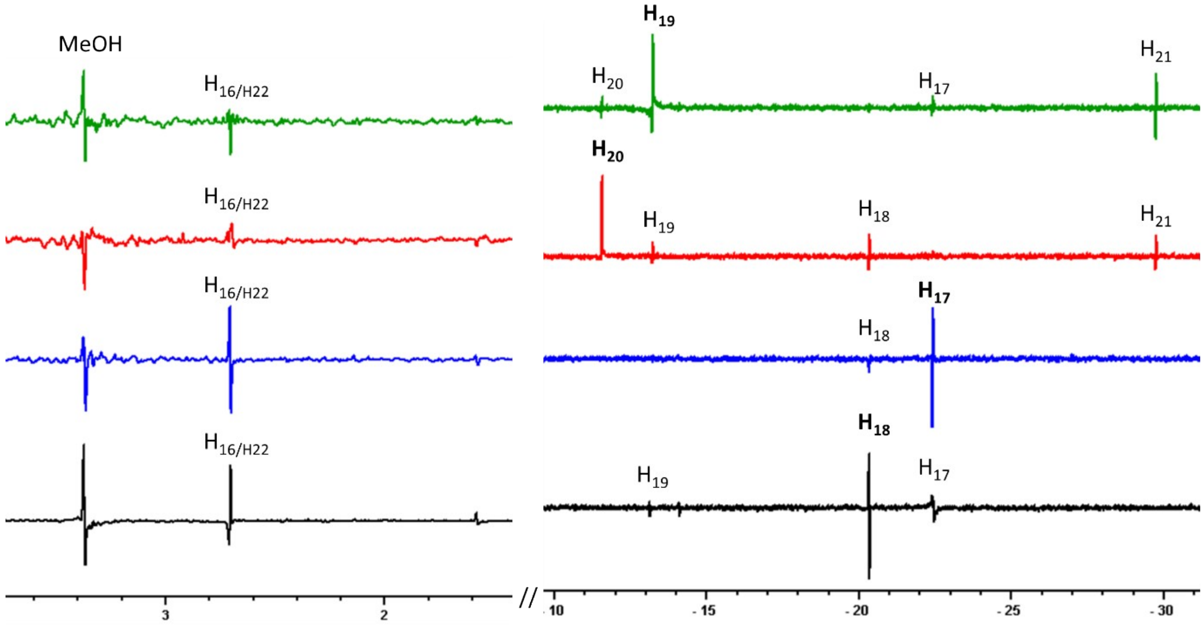

Figure S18: ^{1}H selective COSY in methanol-d_3 at 245 K for a solution of 9. The sites of 9 denoted in bold are selectively excited.

S4 Formation of 7 and 8 from 3

A control reaction involving **1** (5 mM) and H_2 (3 bar) with DMSO (25 mM) in methanol-d_3 or methanol-d_4 forms mixtures of **3** and **5**. NaOMe (50 mM) was then added, and the speciation changes monitored using ^{1}H NMR spectroscopy at 298 K.

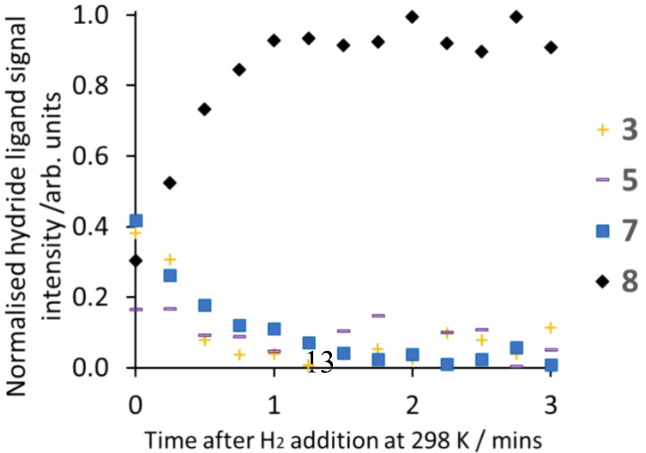

***Figure S19:** Reaction time course profile after NaOMe (50 mM) is added to a mixture of preformed 3 (from a 1 (5 mM), DMSO (25 mM) and H_2 (3 bar) sample in methanol-d$_3$) at 298 K.*

S5 Hyperpolarisation of 8

A sample containing **1** (5 mM), DMSO (25 mM) and NaOMe (50 mM) in methanol-*d*$_4$ was reacted with *p*H_2 at 298 K and the sample shaken at a magnetic field of 65 G for 10 seconds (ALTADENA-like conditions) before being rapidly placed into a 9.4 T NMR spectrometer and examined using a single-scan 45° ^{1}H pulse (Figure S20). The process was repeated at several time intervals after *p*H_2 addition, each time with addition of fresh *p*H_2 and reshaking.

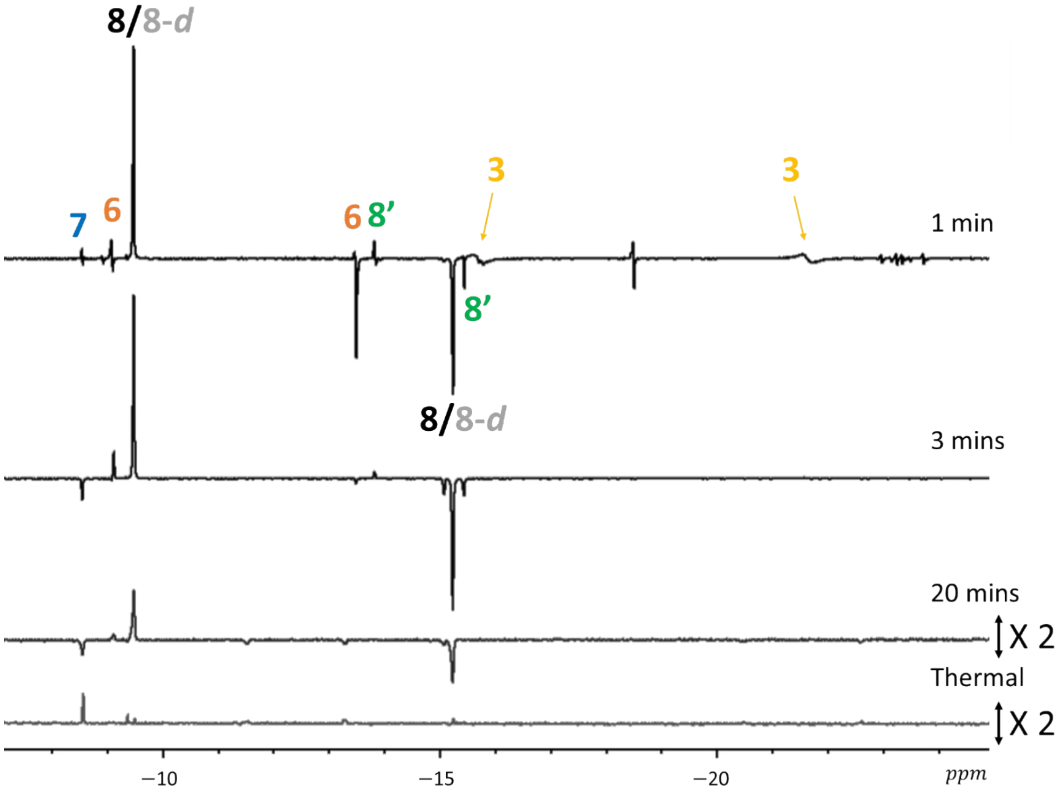

***Figure S20:** Hyperpolarised single scan ^{1}H NMR spectra recorded using a 45° pulse at the indicated reaction time point after the addition of pH_2 to a methanol-d$_4$ solution of 1 (5 mM), DMSO (25 mM) and NaOMe (50 mM) at 298 K. Before each of these NMR spectra were recorded, the sample was shaken with fresh pH_2 for 10 seconds at 65 G to initiate PHIP.*

A sample containing **1** (5 mM), DMSO (25 mM) and NaOMe (50 mM) in DCM-*d*$_2$ was reacted with *p*H_2 at 298 K and the sample shaken at a magnetic field of 65 G for 10 seconds (ALTADENA-like conditions) before being rapidly placed into an 9.4 T NMR spectrometer and examined using a single-scan 45° ^{1}H pulse (Figure S21). The process was repeated at several time intervals after *p*H_2 addition, each time with addition of fresh *p*H_2 and reshaking.

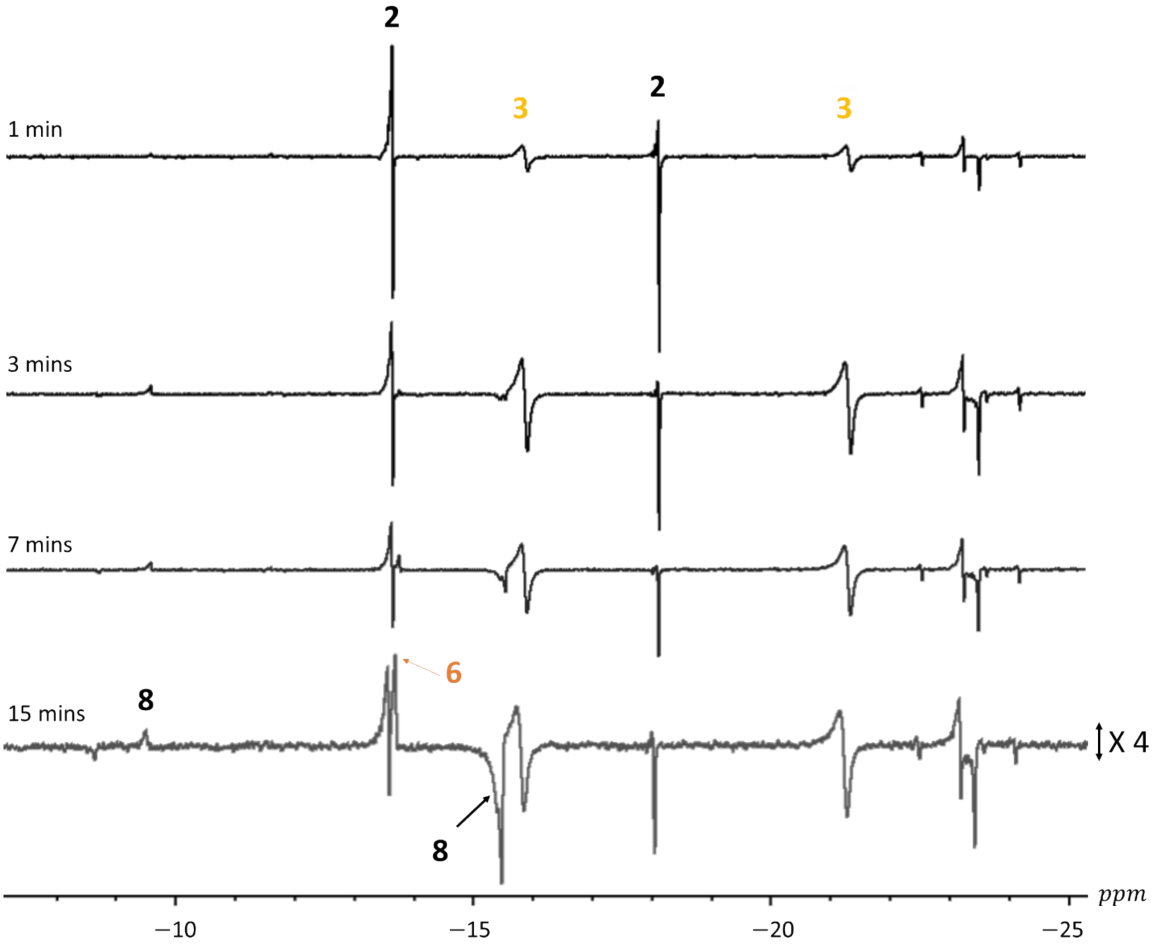

***Figure S21:** a) Hyperpolarised single scan ^{1}H NMR spectra recorded using a 45° pulse at the indicated time interval after addition of pH_2 to 1 (5 mM), DMSO (25 mM) and NaOMe (50 mM) in DCM-d_2 at 298 K at 9.4 T. Before each NMR spectrum was recorded the sample was shaken for 10 seconds at 65 G with fresh pH_2.*

S6 NMR Characterisation of 11 and 12

Further confirmation on the identify of **8** came from the replacement of DMSO with MPSO. As MPSO is chiral, binding leads to two diasteriomeric pairs with inequivalent hydride ligands. Viewing the arrangement of the equatorial sulfoxide relative to its axial partner details how the in plane hydride ligands become distinct. These isomers exist in different proportions due to their diasteriotopic nature,

and nOe connections have ensured their assignment. The sample was formed by allowing **1** (5 mM) to react with MPSO (25 mM) in the presence of NaOMe (50 mM) at 298 K, analogous results were obtained with NaH.

When the reaction was allowed to proceed at 263 K, a series of further resonances, in addition to those of **11** and **12** were observed at *δ* −11.68 (*q*, 6 Hz), −12.08 (*q*, 6 Hz), −14.08 (*q*, 6 Hz) and −14.95 (*q*, 6 Hz). These have relative area 1:1:1:1, and the latter peak overlaps with one for **12**. This is consistent with the detection of the *cis* isomer of **11** in which all four hydride ligands are distinct.

The structure of **11** and **11'** are shown in Figure S22, and their NMR resonances are detailed in Table S7.

11

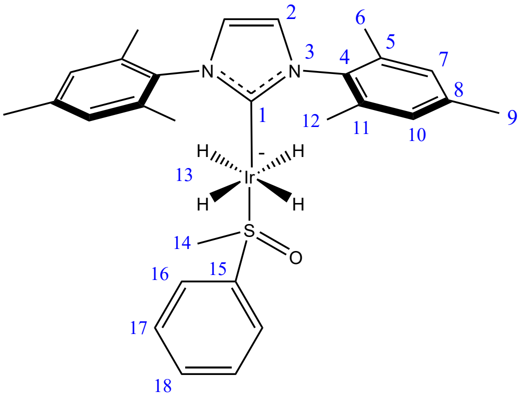

11'

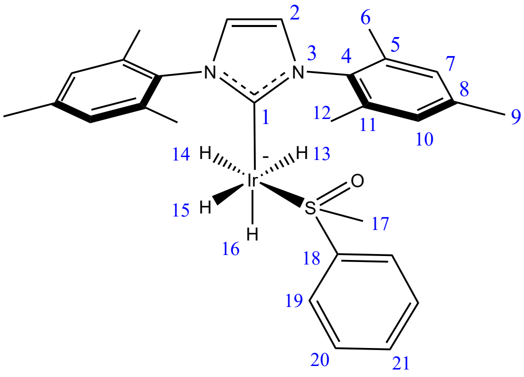

Figure S22: Structure of 11 and 11'.

Table S7: NMR resonances of 11' in toluene-d_8 at 253 K and partial NMR signals for unstable 11. The resonance labels are according to Figure S22.

	11		11'		
**Resonance Number**	**^{1}H / ppm**	**^{13}C / ppm**	**^{1}H / ppm**	**^{13}C / ppm**	**^{15}N / ppm**
**1**		-		-	
**2**	-	-	6.45	119.33	
**3**					197.45
**4**		-		141.27	
**5**		-		136.31	
**6**	-		2.41		
**7**		-		127.96	
**8**		-		135.22	
**9**	-		2.28		
**10**		-		128.18	
**11**		-		137.17	
**12**	2.08	-	2.65	-	
**13**	−7.90 (*s*)		−14.95 (1H, *q* 5 Hz)		
**14**	2.95		−14.21 (1H, *q* 5 Hz)		
**15**		-	−12.13 (1H, *q* 5 Hz)		
**16**	7.82	-	−11.75 (1H, *q* 5 Hz)		
**17**	7.10	-	3.62	-	
**18**	7.04	-		-	
**19**			8.70	127.44	
**20**			7.18	-	
**21**			-	-	

The structure of **12** are shown in Figure S23, and its NMR resonances detailed in Table S8.

$\mathbf{12_{maj}}$

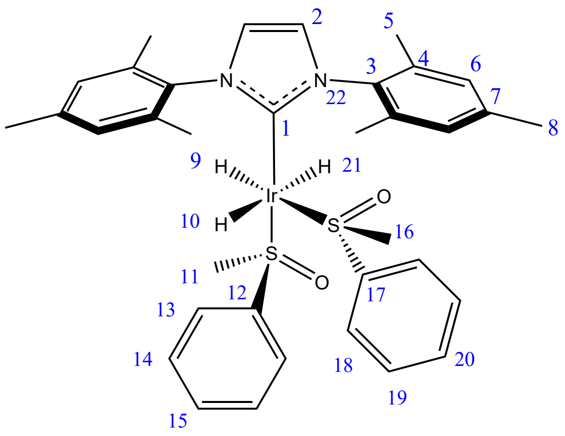

$\mathbf{12_{min}}$

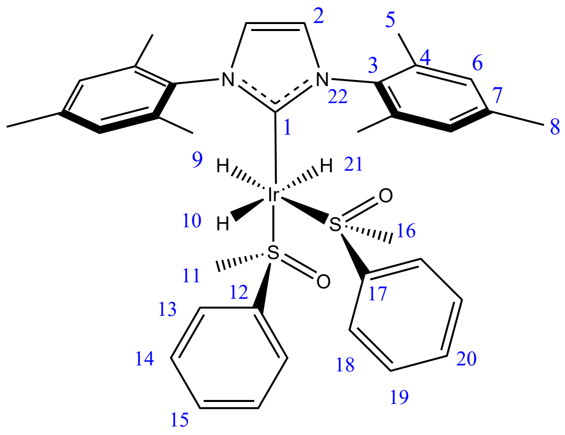

Figure S23: Structure of 12.

Table S8: NMR resonances of 12 in toluene-d_8 at 298 K. The resonance labels are according to Figure S23.

	Major isomer (75%)			*Minor isomer (25%)*		
**Resonance Number**	**^{1}H / ppm**	**^{13}C / ppm**	**^{15}N / ppm**	**^{1}H / ppm**	**^{13}C / ppm**	**^{15}N / ppm**
**1**		159.41			158.72	
**2**	6.31	120.56		6.34	120.79	
**3**		135.15			135.71	
**4**		139.19			139.37	
**5**	2.13, 2.48	18.66, 20.78		2.21, 2.50	19.08, 19.49	
**6**	6.50, 6.88	128.04, 128.83		6.61, 6.88	128.56, 128.53	
**7**		136.79			136.24	
**8**	2.47	19.85		2.20	20.80	
**9**	− 15.03 (*t*, 5 Hz)			− 14.78 (*t*, 5 Hz)		
**10**	− 8.65 (*dd*, 5 Hz, 11 Hz)			− 9.20 (*dd*, 5 Hz and 11 Hz)		
**11**	3.37 (*s*)	67.22		3.26	66.49	
**12**		153.40			-	
**13**	8.08	126.83		8.14	126.04	
**14**	7.12	127.14		7.10	128.09	
**15**	7.05	129.82		6.97	-	
**16**	3.14	57.44		3.62	70.77	
**17**		149.35			-	
**18**	8.29	126.97		7.89	125.89	
**19**	7.06	128.68		6.85	129.36	
**20**	7.05	129.96		6.82	-	
**21**	− 8.79 (*dd*, 5 Hz and 11 Hz)			− 8.41 (*dd*, 5 Hz and 11 Hz)		
**22**			197.04			197.54

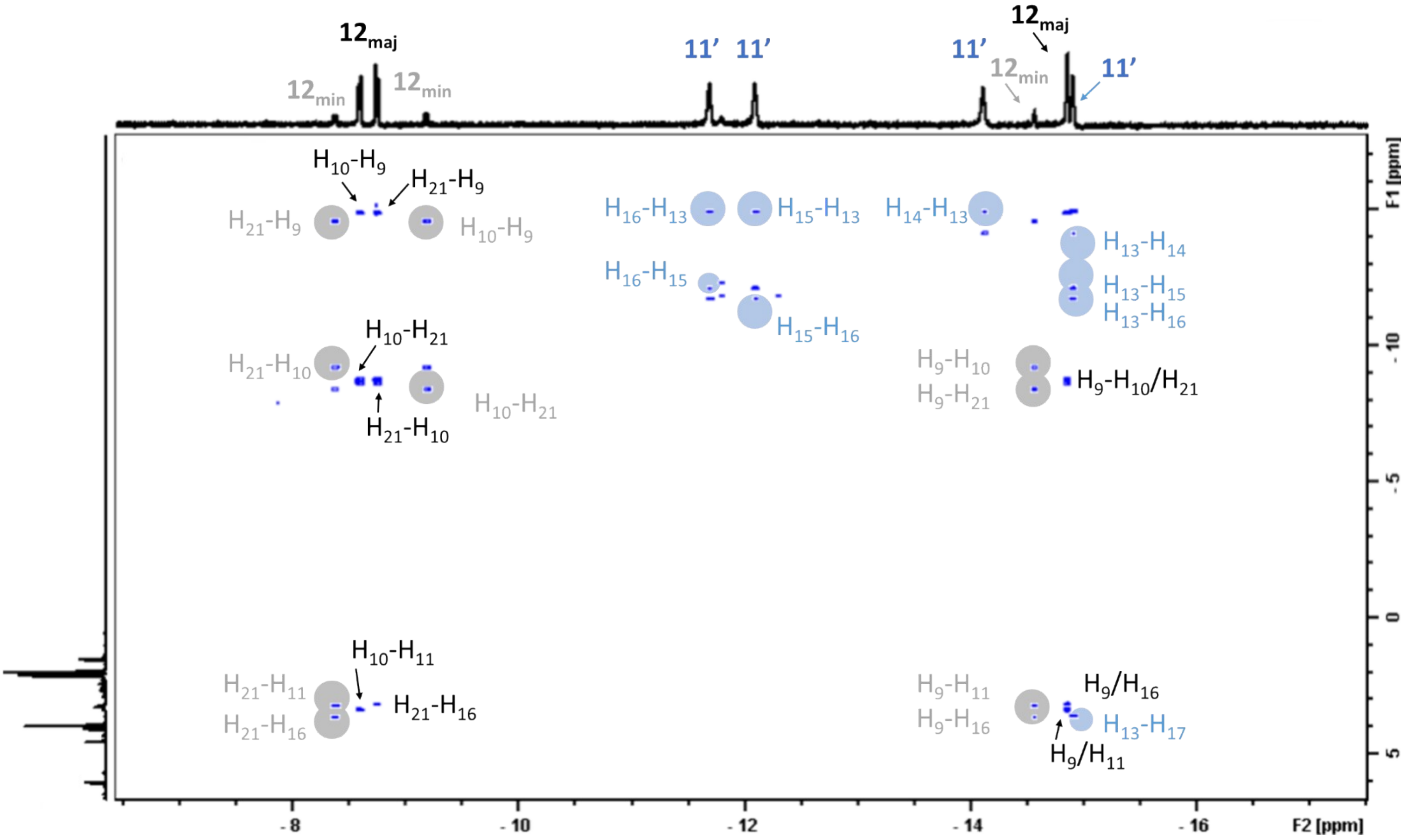

Figure S24: ¹H COSY for a mixture of 11’ and 12 in toluene-d_8 at 253 K

The analogue of **3**, containing MPSO was also characterised by preparing a solution containing **1** (5 mM), MPSO (25 mM) and H_2 (3 bar) in DCM-d_2. The solution was cooled to 243 K for NMR characterisation and its structure and NMR resonances are given in Figure S25 and Table S9 respectively.

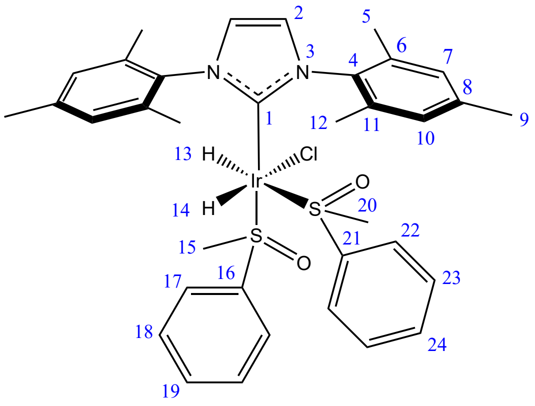

Figure S25: Structure of [Ir(Cl)(H)$_2$(MPSO)$_2$(IMes)].

Table S9: NMR resonances of [Ir(Cl)(H)$_2$(MPSO)$_2$(IMes)] in dichloromethane-d$_2$ at 243 K. The resonance labels are according to Figure S25.

	CD_2Cl_2 at 243 K, major isomer		
Resonance Number	***^1H / ppm***	***^{13}C / ppm***	***^{15}N / ppm***
**1**		152.51	
**2**	6.89	123.03	
**3**			196.60
**4**		137.66	
**5**	2.19	18.85	
**6**		136.20	
**7**	6.83	128.23	
**8**		137.96	
**9**	2.20	20.92	
**10**	6.83	128.23	
**11**		136.20	
**12**	2.19	18.85	
**13**	− 14.83 (*d*, 6 Hz)		
**14**	− 21.79 (*d*, 6 Hz)		
**15**	3.45	51.24	
**16**		150.13	
**17**	7.70	125.21	
**18**	7.54	128.09	
**19**	7.55	131.12	
**20**	3.26	44.56	
**21**		146.30	
**22**	7.22	125.33	
**23**	7.05	127.95	
**24**	7.35	130.95	

S7 2D NMR pulse sequences

S7.1 HMQC

Pulse sequence: hmqcgpqf

```
1 ze
2 d1 do:f2
3 p1 ph1
  d2 pl2:f2 UNBLKGRAD
  p3:f2 ph3
  d0
  p16:gp1
  d16
  p2 ph2
  d13
  p16:gp2
  d16
  d0
  p3:f2 ph4
  d13
  p16:gp3
  d16
  DELTA1
  d12 pl12:f2 BLKGRAD
  go=2 ph31 cpd2:f2
  d1 do:f2 mc #0 to 2 F1QF(caldel(d0, +in0))
exit

ph1=0
ph2=0
ph3=0 2
ph4=0 0 2 2
ph31=0 2 2 0
```

Typical parameters are o1p = 5 ppm (^{1}H) and 100 ppm (^{13}C); SW = 10 ppm (^{1}H) and 200 ppm (^{13}C); AQ = 0.3 s; TD = 2k-4k (F2) and 256-1k (F1); DS = 16; NS = 16; D1 = 1.5 s; d16 = 2 ms; cnst 12 = 145 Hz for direct couplings or 12 Hz for distant couplings. SMSQ10.100 gradient files are used with a 1 ms length (p16) in a ratio of 50%, 30% and 40.1% for the three z gradients used in the pulse sequence. P1 = 8µs and PL1 = − 10.76 dB (^{1}H); p3 = 15 µs and PL2 = − 19.85 (^{13}C). PL12 = − 1.79 dB (decoupling).

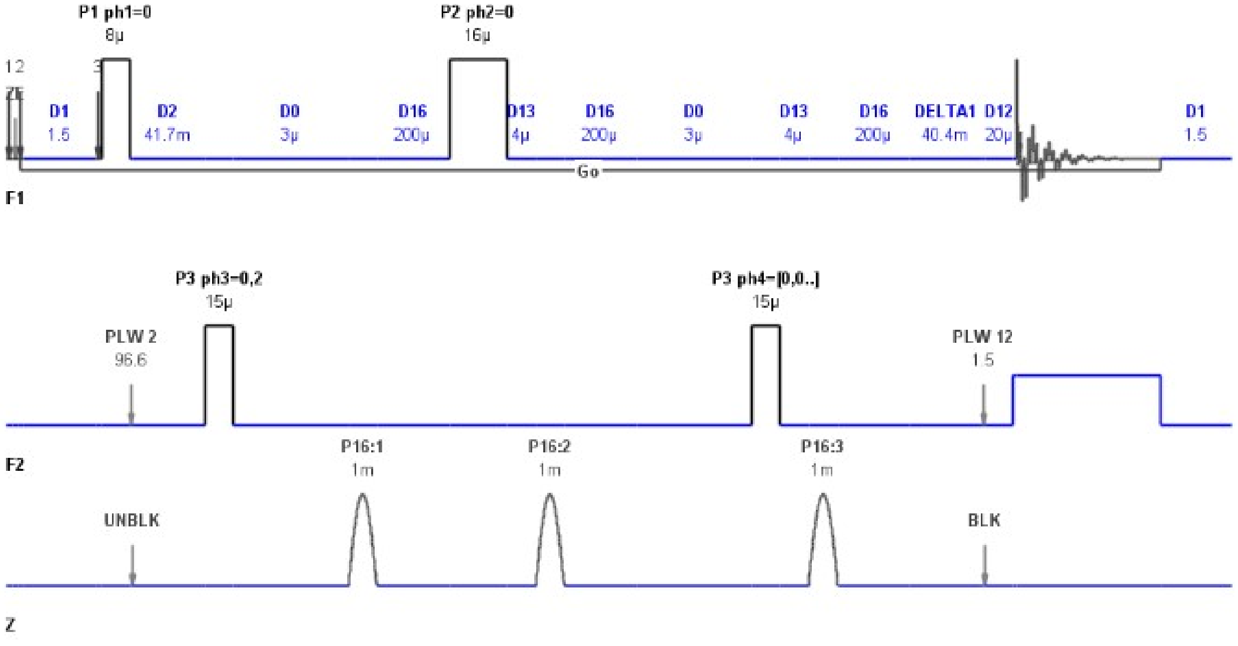

Figure S26: Pulse sequence for ^{13}C-^{1}H HMQC

S7.2 HSQC

Pulse sequence: hsqcetgpsi2

```
1 ze
```

```
  d11 pl12:f2
2 d1 do:f2
3 (p1 ph1)
  d4 pl2:f2
  (center (p2 ph1) (p4 ph6):f2 )
  d4
  p28 ph1
  4u
  (p1 ph2) (p3 ph3):f2
  d0
  d0
  50u UNBLKGRAD
  p16:gp1*EA
  d16
  (p4 ph4):f2
  DELTA
  (center (p1 ph1) (p3 ph4):f2 )
  p19:gp3
  d16
  DELTA2
  (center (p2 ph1) (p4 ph1):f2 )
  DELTA2
  p19:gp3
  d16
  (center (p1 ph2) (p3 ph5):f2 )
  p16:gp4
  d16
  DELTA3
  (center (p2 ph1) (p4 ph1):f2 )
  DELTA3
  p16:gp4
  d16
  (p1 ph1)
  DELTA1
  (p2 ph1)
  4u
  p16:gp2
  d16 pl12:f2
  4u BLKGRAD
  go=2 ph31 cpd2:f2
  d1 do:f2 mc #0 to 2
     F1EA(calgrad(EA) & calph(ph5, +180), caldel(d0, +in0) & calph(ph3, +180) & calph(ph6, +180) & calph(ph31, +180))
exit

ph1=0
ph2=1
ph3=0 2
ph4=0 0 2 2
ph5=1 1 3 3
ph6=0
ph7=0 0 2 2
ph31=0 2 2 0
```

Typical parameters are o1p = 5 ppm (^{1}H) and 100 ppm (^{13}C); SW = 10 ppm (^{1}H) and 200 ppm (^{13}C); AQ = 0.3 s; TD = 2k-4k (F2) and 256-1k (F1); DS = 16; NS = 16; D1 = 1.5 s; cnst 12 = 145 Hz for direct couplings or 12 Hz for distant couplings

SMSQ10.100 gradient files are used in a ratio of 80%, 20.1%, 11% and -5% for the z gradients with indexes 1, 2, 3 and 4 respectively used in the pulse sequence. P16 = 1ms; P19 = 0.6 ms. P1 = 8µs and PL1 = − 10.76 dB (^{1}H); p3 = 15 µs and PL2 = − 19.85 (^{13}C). PL12 = − 1.79 dB (decoupling). D16 = 2 ms.

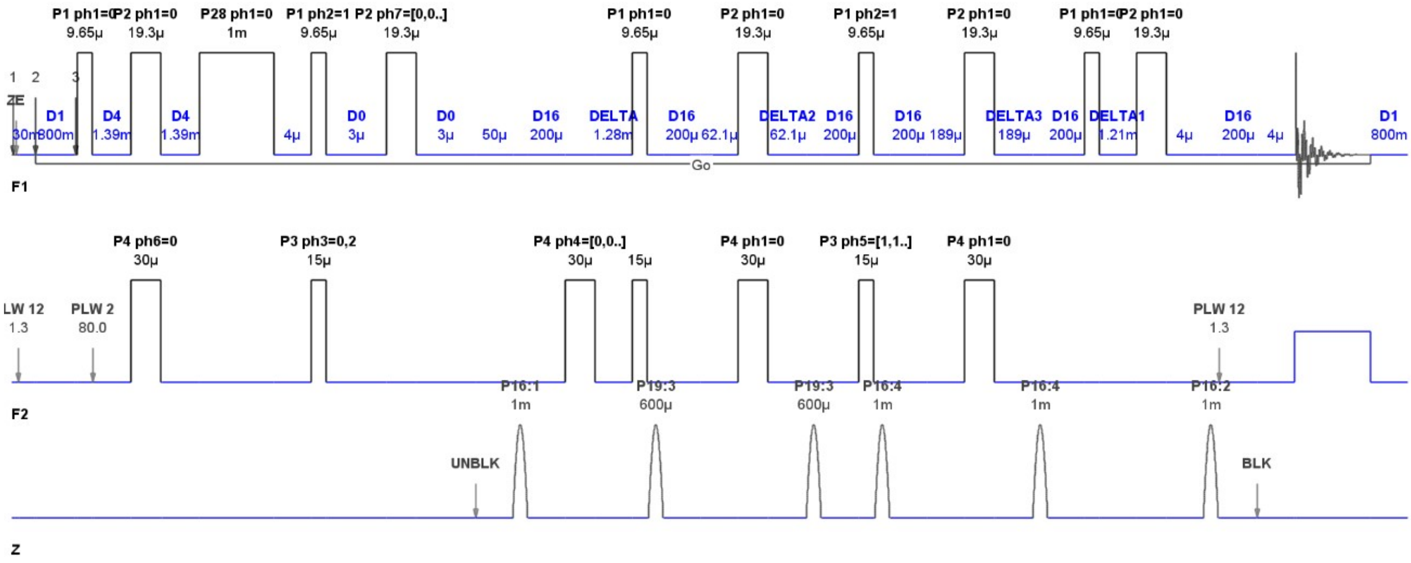

Figure S27: Pulse sequence for ^{13}C-^{1}H HSQC

S7.3 NOESY

Pulse sequence: noesygpph

1 ze

```
2 d1
3 p1 ph1
  d0
  p1 ph2
  TAU
  50u UNBLKGRAD
  p16:gp1
  d16
  3u
  (p2 ph4):f1
  3u
  p16:gp1*-1
  d16
  50u BLKGRAD
  TAU
  p1 ph3
  go=2 ph31
  d1 mc #0 to 2 F1PH(calph(ph1, +90), caldel(d0, +in0))
exit


ph1=0 2
ph2=0 0 0 0 0 0 0 0 2 2 2 2 2 2 2 2
ph3=0 0 2 2 1 1 3 3
ph4=0
ph31=0 2 2 0 1 3 3 1 2 0 0 2 3 1 1 3
```

Typical parameters are o1p = − 10 ppm; SW = 50 ppm; AQ = 0.1-0.2 s; TD = 2k-4k (F2) and 256-1k (F1); DS = 32; NS = 8; D1 = 2 s; d8 = 0.3. SMSQ10.100 gradient files are used with a 1 ms (p16) length in a ratio of 40%. P1 = 10.25 µs and PL1 = − 10.21 dB

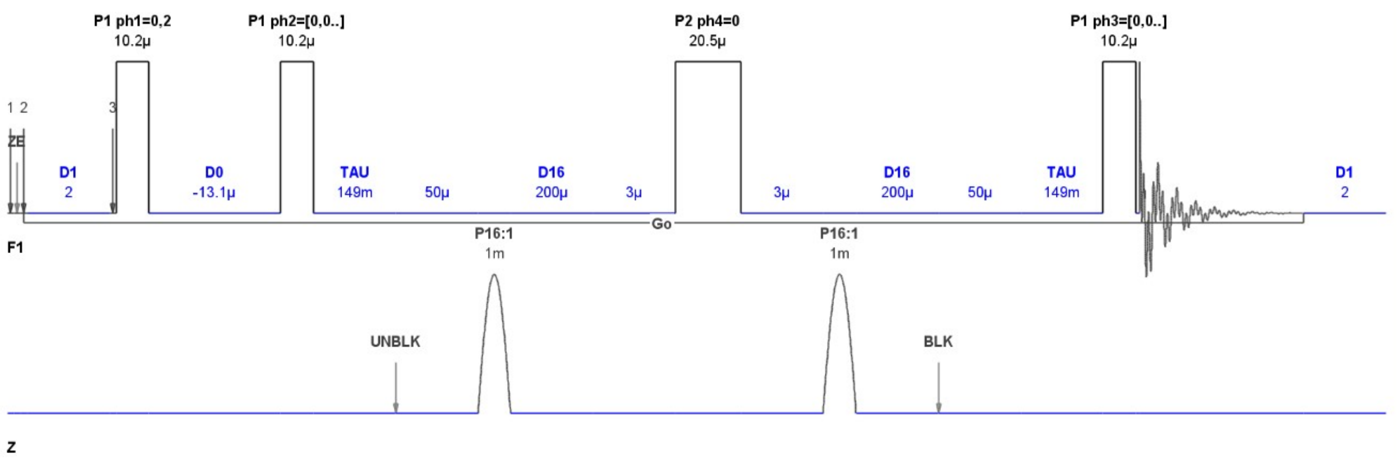

Figure S28: Pulse sequence for NOESY

S7.4 COSY

Pulse sequence: cosygpppqf

```
1 ze
2 d11

3 d12 pl10:f1
  p17 ph3
  p17*2 ph4
  d1 pl1:f1

  p1 ph1
  d0
  50u UNBLKGRAD
  p16:gp1
  d16
  p0 ph2
  d13
  p16:gp1
  d16
  4u BLKGRAD
  go=2 ph31
  d11 mc #0 to 2 F1QF(caldel(d0, +in0))
exit


ph1=0 2
ph2=0 0 2 2
ph3=0
ph4=1
ph31=0 2
```

Typical parameters are o1p = 5 ppm; SW = 10 ppm; AQ = 0.3 s; TD = 2k (F2) and 512 (F1); DS = 16; NS = 8; D1 = 2 s. SMSQ10.100 gradient files are used with a 1 ms (p16) length in a ratio of 10%. P1 = 10.25 µs and PL1 = − 10.21 dB (^{1}H); d16 = 2 ms.

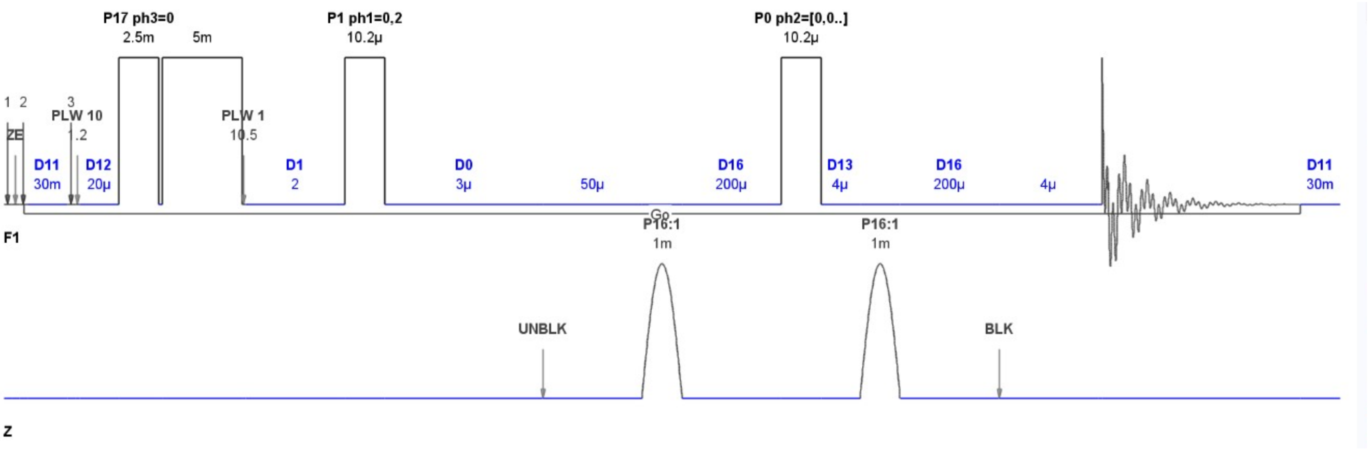

Figure S29: Pulse sequence for COSY

S7.5 Selective NOESY

Pulse sequence: selnogp

```
1 ze
2 30m
  20u pl1:f1
  d1
  50u UNBLKGRAD
  (p1 ph1):f1
  3u
  p16:gp1
  d16 pl0:f1
  p12:sp2:f1 ph2:r
  3u
  p16:gp1
  d16 pl1:f1
  (p1 ph3):f1
  d20
  p16:gp2
  d16
  3u
  (p2 ph4):f1
  3u
  p16:gp2*-1
  d16
  d20
  (p1 ph5):f1
  4u BLKGRAD
  go=2 ph31
  30m mc #0 to 2 F0(zd)
exit

ph1=0 2
ph2=0 0 1 1 2 2 3 3
ph3=0
ph4=0
ph5=0
ph31=0 2 2 0
```

Typical parameters are o1p = peak of interest; SW = 65 ppm; AQ = 1 s; TD = 65k; DS = 4; NS = 32+; D1 = 2 s; d8 = 0.01, p12 = 80,000 µs. SMSQ10.100 gradient files are used with a 1 ms (p16) length in a ratio of 15% and 40% for the gradients with indexes 1 and 2 respectively. P1 = 9.65 µs and PL1 = − 10.79 dB; d16 = 2 ms.

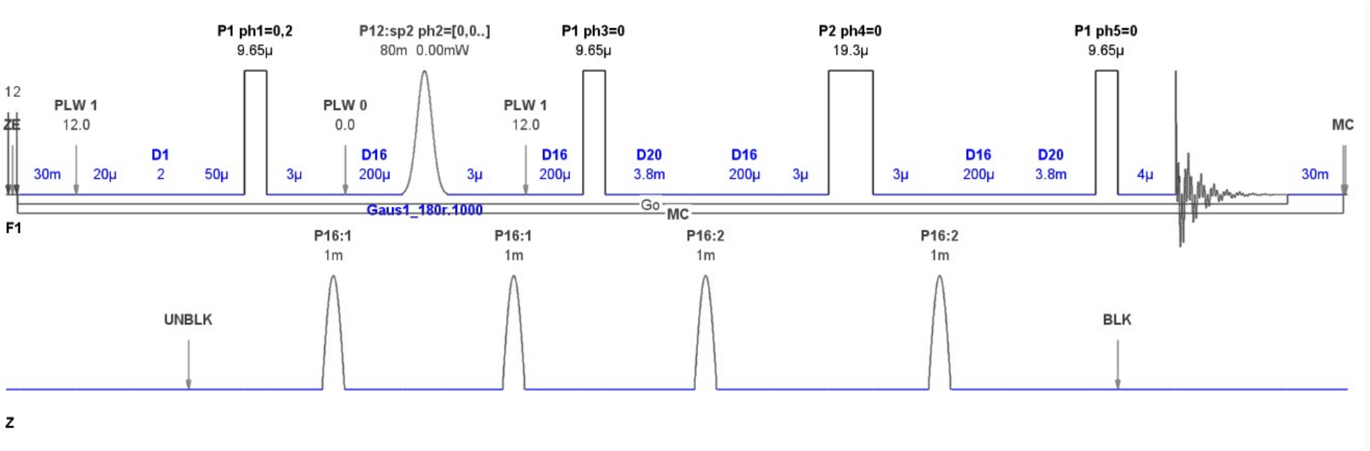

Figure S30: Pulse sequence for selective NOESY

S7.6 Selective COSY

Pulse sequence: selcogp

```
1 ze
2 30m
  20u pl1:f1 BLKGRAD
  d1
  50u UNBLKGRAD
  (p1 ph1):f1
  3u
  p16:gp1
  d16 pl0:f1
  p12:sp2:f1 ph2:r
  3u
  p16:gp1
  d16
  d4 pl1:f1
  (p2 ph3):f1
  d4
  (p1 ph4):f1
  go=2 ph31
  30m mc #0 to 2 F0(zd)
  20u BLKGRAD
exit

ph1=0 2
ph2=0 0 1 1 2 2 3 3
ph3=0
ph4=1
ph31=0 2 2 0
```

Typical parameters are o1p = peak of interest; SW = 65 ppm; AQ = 1 s; TD = 40k; DS = 4; NS = 32+; D1 = 2 s; p12 = 80,000 µs. SMSQ10.100 gradient files are used with a 1 ms (p16) length in a ratio of 15%. D4 = 0.05 s, P1 = 9.65 µs and PL1 = − 10.79 dB; d16 = 2 ms.

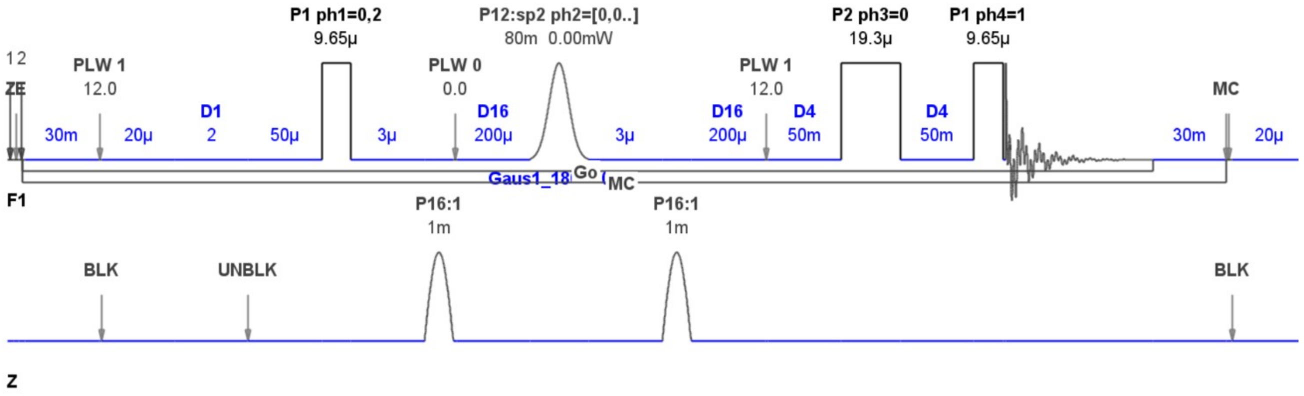

Figure S31: Pulse sequence for selective COSY

8 References

1. Tickner, B. J., Lewis, J. S., John, R. O., Whitwood, A. C. & Duckett, S. B. Mechanistic insight into novel sulfoxide containing SABRE polarisation transfer catalysts. *Dalton Trans.* 48, 15198–15206 (2019).
2. Tickner, B. J., Whitwood, A. C., Condon, C., Platas-Iglesias, C. & Duckett, S. B. Trapping Highly Reactive Metal (H) 2 (η2-H2) species to form Trihydride Complexes and Clusters. *Eur. J. Inorg. Chem.* e202400397 (2024).
